# Dissecting expression profiles of gastric precancerous lesions and early gastric cancer to explore crucial molecules in intestinal‐type gastric cancer tumorigenesis

**DOI:** 10.1002/path.5434

**Published:** 2020-05-27

**Authors:** Yajing Zhang, Xi Wu, Chengli Zhang, Jiaqi Wang, Guijun Fei, Xuebing Di, Xinghua Lu, Lin Feng, Shujun Cheng, Aiming Yang

**Affiliations:** ^1^ State Key Laboratory of Molecular Oncology, Department of Etiology and Carcinogenesis National Cancer Center/National Clinical Research Center for Cancer/Cancer Hospital, Chinese Academy of Medical Sciences and Peking Union Medical College Beijing PR China; ^2^ Department of Gastroenterology Peking Union Medical College Hospital, Chinese Academy of Medical Sciences and Peking Union Medical College Beijing PR China; ^3^ Department of Oncology The Affiliated Hospital of Yangzhou University, Yangzhou University Yangzhou PR China

**Keywords:** gastric tumorigenesis, gastric precancerous lesions, early gastric cancer, tumor hallmarks, stemness, immune infiltration, prognosis

## Abstract

Intestinal‐type gastric cancer (IGC) has a clear and multistep histological evolution. No studies have comprehensively explored gastric tumorigenesis from inflammation through low‐grade intraepithelial neoplasia (LGIN) and high‐grade intraepithelial neoplasia (HGIN) to early gastric cancer (EGC). We sought to investigate the characteristics participating in IGC tumorigenesis and identify related prognostic information within the process. RNA expression profiles of 94 gastroscopic biopsies from 47 patients, including gastric precancerous lesions (GPL: LGIN and HGIN), EGC, and paired controls, were detected by Agilent Microarray. During IGC tumorigenesis from LGIN through HGIN to EGC, the number of activity‐changed tumor hallmarks increased. LGIN and HGIN had similar expression profiles when compared to EGC. We observed an increase in the stemness of gastric epithelial cells in LGIN, HGIN, and EGC, and we found 27 consistent genes that might contribute to dedifferentiation, including five driver genes. Remarkably, we perceived that the immune microenvironment was more active in EGC than in GPL, especially in the infiltration of lymphocytes and macrophages. We identified a five‐gene signature from the gastric tumorigenesis process that could independently predict the overall survival and disease‐free survival of GC patients (log‐rank test: *p* < 0.0001), and the robustness was verified in an independent cohort (*n* > 300) and by comparing with two established prognostic signatures in GC. In conclusion, during IGC tumorigenesis, cancer‐like changes occur in LGIN and accumulate in HGIN and EGC. The immune microenvironment is more active in EGC than in LGIN and HGIN. The identified signature from the tumorigenesis process has robust prognostic significance for GC patients. © 2020 The Authors. *The Journal of Pathology* published by John Wiley & Sons Ltd on behalf of Pathological Society of Great Britain and Ireland.

## Introduction

Gastric cancer (GC) is the fifth most frequently diagnosed cancer and the third leading cause of cancer‐related death worldwide [1]. Gastric adenocarcinomas can be categorized into mainly intestinal and diffuse types according to the Lauren classification [2, 3]. Intestinal‐type GC (IGC) has a clear and multistep histological evolution that starts with inflammation and progresses through atrophy, intestinal metaplasia, gastric precancerous lesion [GPL, including low‐grade intraepithelial neoplasia (LGIN) and high‐grade intraepithelial neoplasia (HGIN)] to early GC (EGC) and then advanced GC (AGC) [4]. Most of the previous studies on GC have highlighted differences between AGC and the adjacent mucosa from patients undergoing gastrectomy [5, 6]. Although the genomic signature of primary GC has been well characterized, there are still many unidentified mechanisms underlying different steps of the carcinogenic cascade, such as no specific mutation pattern during cancerization of intestinal‐type GC, which restricts the diagnosis and treatment of GC [7]. Although some studies on premalignant gastric mucosa and EGC have been performed [8, 9, 10, 11, 12], no comprehensive molecular expression profiles interpreting the intact process of gastric tumorigenesis from GPL to EGC are available.

Gene expression signatures can be used as molecular predictors of overall survival (OS) and relapse of GC independent of TNM stage [13, 14, 15, 16]. However, GC is highly heterogeneous in both phenotype and genotype, causing the gene signatures identified as prognosis predictors to depend greatly on the training set, which might limit the validity and the reproducibility in other cohorts, thereby emphasizing the importance of exploring molecular alterations in concert [14]. The process of tumorigenesis and progression provides an appropriate model to identify consistent genes to improve the diagnosis and prognosis prediction.

In the present study, we used LGIN, HGIN, EGC, and their paired inflammation controls to represent gastric tumorigenesis. We characterized the changes in gene expression, biological processes, tumor hallmark activities, stemness, and immune microenvironment during gastric tumorigenesis. Furthermore, we identified the gene signature from the gastric tumorigenesis and progression process to construct a risk model to predict OS and relapse of GC patients.

## Materials and methods

### Patients and samples

Forty‐seven patients with GPL or EGC were identified from the Department of Gastroenterology, Peking Union Medical College Hospital (PUMCH) from 2011 to 2015. Endoscopic biopsies were performed in each case according to the following parallel protocol: specimens for pathology were obtained with forceps from the serious part of the lesion; then an additional specimen was taken from the same spot in the lesion for our study. After systematic inspection with enhanced imaging techniques, the relatively normal area was localized and then two specimens were obtained from the same spot, sent for pathology and study control, respectively. In total, 94 specimens were obtained. Pathological results were confirmed by two independent pathologists (Linlin Guo and Weixun Zhou). Gastroscopic biopsies were rapidly immersed in RNAlater® solution (Cat No AM7021; Invitrogen, Waltham, MA, USA) and transferred to −80 °C after overnight storage at 4 °C. According to the *WHO Classification of Tumors of the Digestive System* for pathological diagnosis [17], the samples were grouped into four categories: chronic gastritis, LGIN, HGIN, and EGC. Tissues with a pathological diagnosis of inflammation based on the Updated Sydney System [18] were used as controls. Tissues that were diagnosed as atrophic gastritis and intestinal metaplasia were excluded. This study was approved by the Ethics Committee of PUMCH and received institutional approval [institutional review board (IRB) number: B222]. All the patients provided written informed consent, and the experiments were performed in accordance with the World Medical Association Declaration of Helsinki Ethical Principles for Medical Research [19].

### Microarray expression profiling and data normalization

Total RNA was extracted from frozen tissues using an RNeasy Mini Kit (Cat No 74106; Qiagen, Hilden, Germany) according to the manufacturer's instructions. Subsequently, purified RNA samples were labeled and hybridized to an Agilent SurePrint G3 Human GE v2 8 × 60K Microarray (Agilent Technologies, Santa Clara, CA, USA) according to the manufacturer's instructions.

The raw data were normalized using GeneSpring GX software, version 11.5 (Silicon Genetics, Redwood City, CA, USA). The expression value for a particular gene that was mapped by multiple probes was determined as the probe with the highest median expression value across all samples [20, 21]. The raw data and processed data have been deposited in the National Center for Biotechnology Information (NCBI) Gene Expression Omnibus (GEO) database with accession number GES130823.

### Data collection

TCGA RNA sequence level 3 data [raw counts and RNASeq by Expectation Maximization (RSEM) normalized read counts] of 415 stomach adenocarcinomas (TCGA STAD), 35 normal stomach tissues, and the related clinical information were obtained from the cBioPortal for Cancer Genomics database (http://www.cbioportal.org/). The raw data of two human GC mRNA microarray studies with prognostic information (accession numbers GSE62254 and GSE15460; sample size > 200) were downloaded from the GEO database. These two mRNA microarray datasets were based on the same platform [HG‐U133A Plus2 (GPL570) platform].

### Identification of differentially expressed genes and functional enrichment

Paired or unpaired Student's *t*‐tests were conducted to identify differentially expressed genes (DEGs) between lesions and paired controls or among lesions. DEGs from stomach adenocarcinoma data of TCGA were obtained using the Bioconductor package ‘DEseq2’. Protein coding genes with a false discovery rate (FDR) < 0.05 and a fold‐change (FC) > 1.5 were regarded as DEGs. The R package ‘ClusterProfiler’ was applied for Gene Ontology (GO) and Kyoto Encyclopedia of Genes and Genomes (KEGG) pathway enrichment analyses.

### Hallmark activity assessment

Gene sets related to ten hallmarks of cancer were downloaded from Gene Set Enrichment Analysis (GSEA; http://software.broadinstitute.org/gsea/index.jsp) [22, 23]. Gene set variation analysis (GSVA) [24] was applied to estimate the activity scores of tumor hallmarks using these gene sets. All genes related to each hallmark were employed to score the activity of this hallmark in the control tissues, which was considered the baseline activity for each lesion stage.

### Stemness index production and driver gene expression

The stemness indices were computed referring to the method presented in the TCGA PanCanAtlas Stemness project that was contraposed to mRNA expression (mRNAsi; https://bioinformaticsfmrp.github.io/PanCanStem_Web/) [25].

A list of 299 cancer driver genes from the PanCancer Project was downloaded, and their expression levels were compared between lesions and paired controls. Driver genes with FDR < 0.05 and FC > 1.5, or FC > 2 were considered as differentially expressed driver genes.

### Immune cell infiltration assessment

Unsupervised hierarchical clustering was performed to cluster lesions with 46 immune markers [26]. GSVA and CIBERSORT were run with the validated LM22 gene signature matrix of leukocytes to measure the immune infiltration score, relative fraction, and absolute infiltration of 22 immune cell types [24, 27] (https://cibersort.stanford.edu/). Five previously reported representative immune gene signatures were imitated by GSVA [28], including ‘CSF1_response’ that represents the activation of macrophages/monocytes [29], ‘IFNγ_response’ [30], ‘LIexpression_score’ that represents overall lymphocyte infiltration [31], ‘TGFβ_response’ [32] and ‘Wound Healing’ [33].

TCGA STAD mRNA expression profiles were used to estimate the correlation between gene expression and immune cell infiltration, which was executed by TIMER [34] (https://cistrome.shinyapps.io/timer/).

### Development of a five‐gene risk scoring system for survival analysis

The combination (*n* = 548) of the two GC mRNA microarray datasets was designated as the training dataset. The Least Absolute Shrinkage and Selection Operator (LASSO) Cox regression model that was performed by R package ‘glmnet’ was used to narrow down the 22 consistent DEGs (coDEGs) to select the most useful prognostic markers, in which the training dataset was subsampled and the tuning parameter lambda was determined according to the expected generalization error estimated from leave‐one‐out cross‐validation [35, 36]. We then constructed a signature using the expression value of the identified genes and weighted by the regression coefficient. Finally, a five‐gene signature was obtained under the value of tuning parameter lambda that gives minimum mean cross‐validated error. The risk score of the signature was then calculated using the risk score formula:

$$\text{Risk score}=\left(0.151\times \text{expression of}\;\text{GADD}45B\right)+\left(-0.0754\times \text{expression of}\;\text{LAMP}3\right)+\left(-0.0994\times \text{expression of}\;\text{PLEKHS}1\right)+\left(0.0597\times \text{expression of}\;\mathrm{RGN}\right)+\left(0.266\times \text{expression of}\;\text{TIMP}1\right)$$

X‐tile plot was utilized to select the optimum cutoff score of the training dataset to dichotomize patients into a high‐risk group or a low‐risk group [35, 37]. Kaplan–Meier survival analysis and the log‐rank test were used to evaluate the prognostic difference of the signature between two groups [38]. Multivariate Cox proportional hazard regression analysis was used to evaluate independent prognostic factors.

Harrell's concordance index (C‐index) was used to assess and compare the discrimination and prognostic accuracy of the prognostic signatures [35, 39].

### Statistical analysis

Pearson's chi‐squared test or the Kruskal–Wallis chi‐squared test was used to test the variation of clinical data. Paired Student's *t*‐tests or Wilcoxon signed rank tests were used to calculate differences between paired samples, and an unpaired Student's *t*‐test or Mann–Whitney test was used to calculate differences between unpaired samples. Pearson's product–moment correlation test was used to estimate correlations between gene expression and stemness indices.

## Results

### Changes in biological functions during gastric tumorigenesis

**Figure 1:**
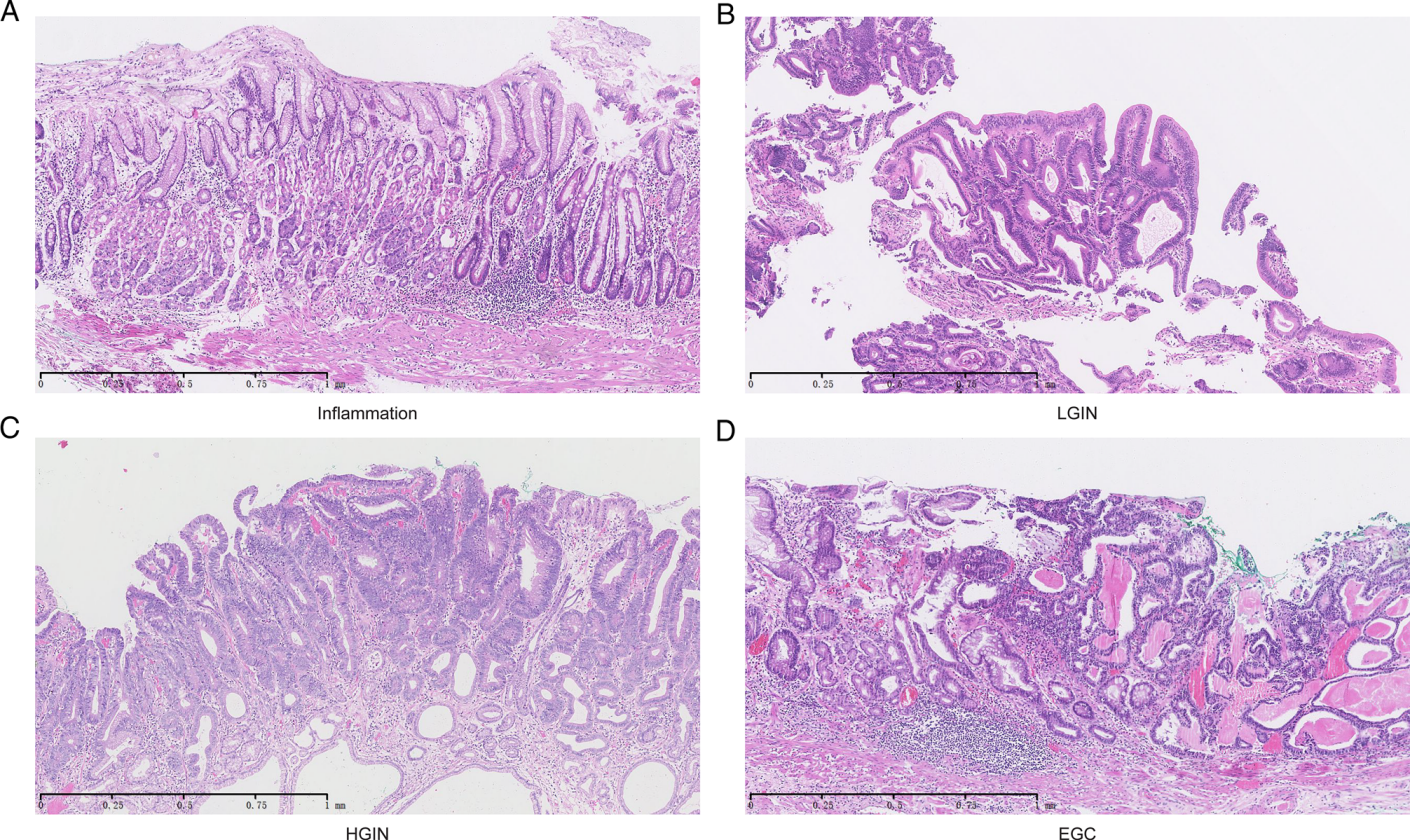
Micrographs of H&E‐stained tissues showing various stages of intestinal‐type GC. H&E staining showing (A) inflammation, (B) LGIN, (C) HGIN, and (D) EGC. Original magnification: ×50 in A–D.

**Figure 2:**
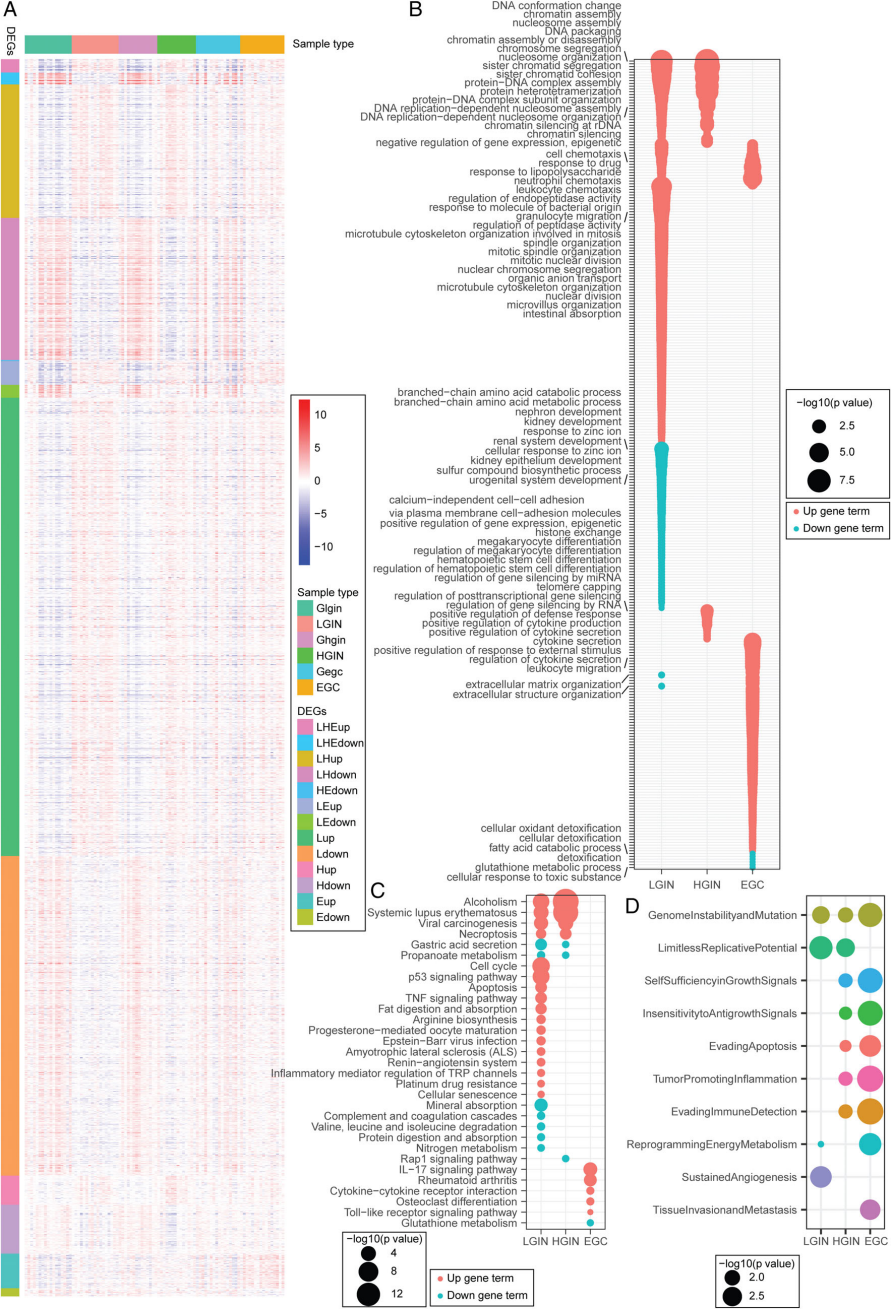
DEGs, GO, and KEGG enrichment results of DEGs and difference of tumor hallmark activities in paired sample groups. (A) Heatmap exhibits DEGs in paired LGIN, HGIN, and EGC group samples. Glgin, Ghgin and Gegc: paired control of LGIN, HGIN, and EGC, respectively; up: up‐regulated DEGs; down: down‐regulated DEGs; LHE: LGIN, HGIN, and EGC common DEGs; LH: LGIN and HGIN common DEGs; HE: HGIN and EGC common DEGs; LE: LGIN and EGC common DEGs; L, H, and E: LGIN‐, HGIN‐, and EGC‐specific DEGs, respectively. (B) GO analysis in terms of biological process and (C) KEGG enrichment of DEGs in LGIN, HGIN, and EGC. UpGeneTerm and DownGeneTerm: up‐regulated and down‐regulated DEGs enrichment results, respectively. (D) Bubble plot shows activities changed tumor hallmarks between LGIN, HGIN, EGC, and their paired control samples, respectively. An FDR adjusted *P* value of less than 0.05 was considered to be statistically significant. In B–D, the size of bubble represents −log_10_(FDR).

We collected 47 paired LGIN, HGIN or EGC tissues and matched inflammation controls (Figure 1 and supplementary material, Table S1). No significant differences were found among the samples in terms of gender (*p* = 0.370) or age (*p* = 0.552) (supplementary material, Table S2). We detected the whole‐genome RNA expression profile of each sample. Through differential gene expression analyses, we found many DEGs between LGIN, HGIN, EGC, and their paired controls (defined as L. DEGs, H. DEGs and E. DEGs, respectively). Most of the H. DEGs and E. DEGs were already present among the L. DEGs (Figure 2A and supplementary material, Figure S1A,B), indicating the presence of cancer‐like genetic alterations in LGIN. Simultaneously, the results showed no DEGs between LGIN and HGIN. The DEGs between EGC and LGIN or HGIN were similar, indicating that the gene expression profiles of GPL were more similar to each other than they were to that of EGC despite the fact that HGIN and EGC are more similar in morphology (supplementary material, Figure S1C–E).

To identify biological behavior changes during gastric tumorigenesis, GO and KEGG enrichment analyses of the three aforementioned DEG groups (L. DEGs, H. DEGs, and E. DEGs) were performed. The up‐regulated DEGs mainly showed enrichment for mitosis in LGIN; for mitosis, cell adhesion in HGIN; and for immune response in EGC. However, the down‐regulated DEGs mainly showed enrichment for metabolism, development, and gastric acid secretion in LGIN; for the Rap1 signaling pathway and gastric acid secretion in HGIN; and for detoxification, metabolism in EGC (Figure 2B,C). Hence, during gastric tumorigenesis, molecular expression and biological functions are substantially changed in LGIN relative to the control gastric mucosa [40].

To further describe the process behind the development of malignant phenotypes during gastric tumorigenesis, we evaluated the activities of ten tumor hallmarks. When we scored with DEGs, the number of hallmarks with significant changes in activity gradually increased from LGIN to HGIN to EGC. The earliest different hallmarks generally included genome instability and mutation and limitless replicative potential, while tissue invasion and metastasis seemed to appear at a later stage, such as EGC tissues in our data (Figure 2D).

### Appearance of cancer‐like changes in LGIN

**Figure 3:**
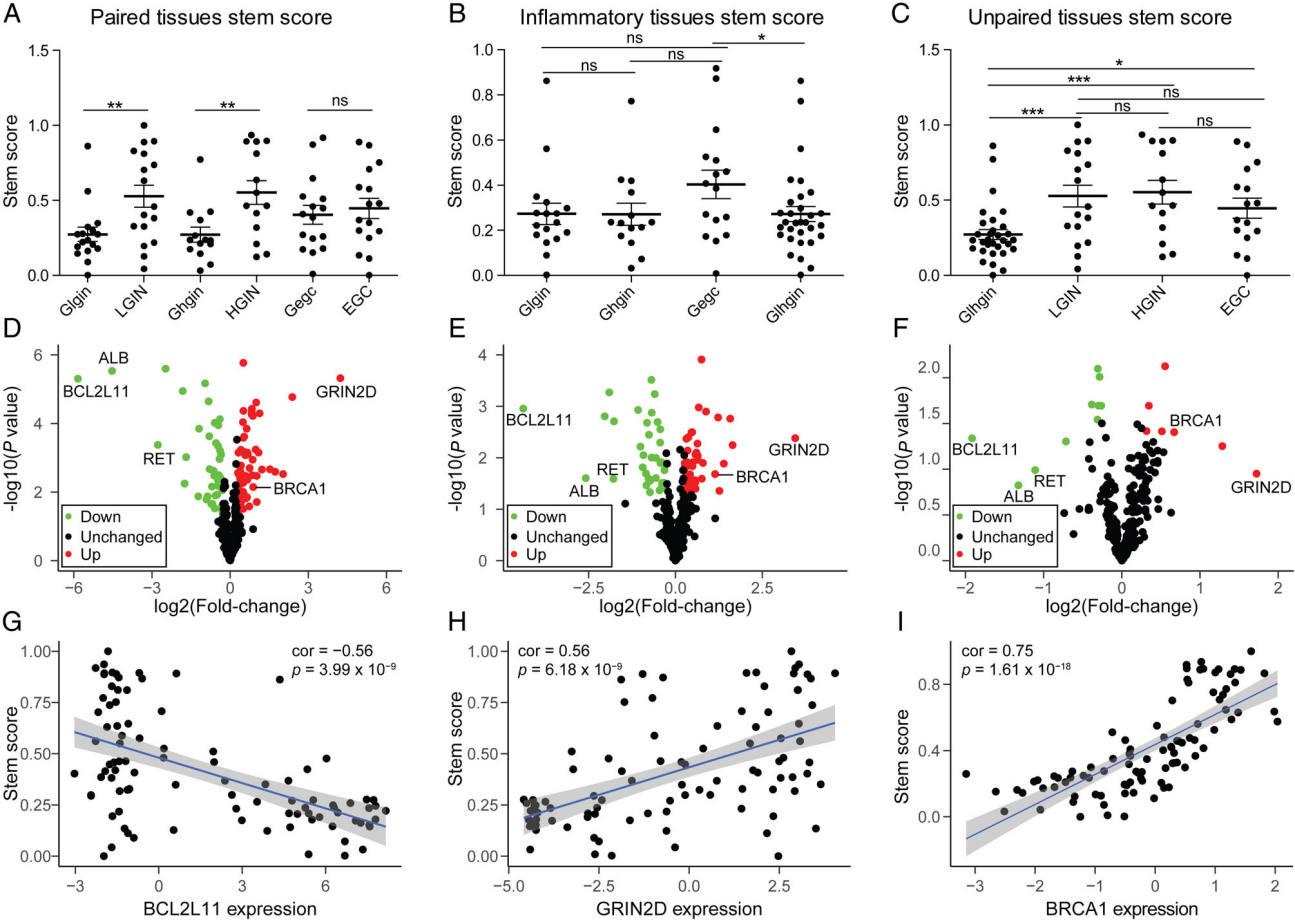
Stemness and expression of 299 pan‐cancer driver genes in different disease stage tissues. (A–C) Stem scores of different group samples. Glhgin represents the control samples of LGIN and HGIN. (D–F) Differential expression of 299 pan‐cancer driver genes in (D) LGIN, (E) HGIN, and (F) EGC relative to their paired control samples. Driver genes with *p* < 0.05 and fold‐change > 1.5, or fold‐change > 2 were identified as up driver genes (red dots). Driver genes with *p* < 0.5 and fold‐change < 1/1.5, or fold‐change < 1/2 were identified as down driver genes (green dots) and other genes were assumed as unchanged driver genes (black dots). (G–I) Correlation of stem scores and three consistent changed driver genes in LGIN, HGIN, and EGC. *p* < 0.05 was considered to be statistically significant. *p < 0.05; **p < 0.01; ***p < 0.001; ns: no significance. Bars show mean ± SEM.

According to the results of the biological function enrichment and tumor hallmark activity analyses, gastric epithelial cells gained limitless replicative potential and impaired gastric acid secretion, which are closely related to stem cell behaviors. To further explore functional changes during gastric tumorigenesis, we characterized the stem‐cell‐like features of different stages by stem score [25]. The stem scores of LGIN and HGIN were significantly higher than those of their paired controls (*p* < 0.05), while there was no difference between EGC and paired control tissues (*p* > 0.05, Figure 3A). In addition, there were no differences in stemness among LGIN, HGIN, and EGC (*p* > 0.05, Figure 3C). However, the stem scores of EGC controls, LGIN, HGIN, and EGC were all significantly higher than those of the controls of LGIN and HGIN (*p* < 0.05, Figure 3B,C) [12]. These results indicated that morphologically normal tissues around EGC have already changed at the molecular level in terms of some basic biological properties, which might be influenced by the tumor and tumor microenvironments.

Furthermore, to reveal the molecular contributions to the stem score changes, we detected the expression of 299 driver genes. We found that the expression levels of *BCL2L11*, *RET*, and *ALB* were significantly reduced in LGIN, HGIN, and EGC compared with their paired controls, and that the expression levels of *GRIN2D* and *BRCA1* were significantly increased (Figure 3D–F). These results provided a more in‐depth explanation at the RNA level that these five driver genes play important roles in gastric tumorigenesis.

We estimated the correlation between the expression of the five common driver genes and the stem scores. All the up‐regulated genes were significantly positively correlated with the stem scores, and all the down‐regulated genes were significantly negatively correlated with the stem scores (|cor| > 0.35, *p* < 0.0001, Figure 3G–I and supplementary material, Figure S2). These results indicated that these genes are important in the dedifferentiated oncogenic phenotype of gastric epithelial cells and that dedifferentiation occurs in LGIN.

### More active immune microenvironment in EGC compared with GPLs

**Figure 4:**
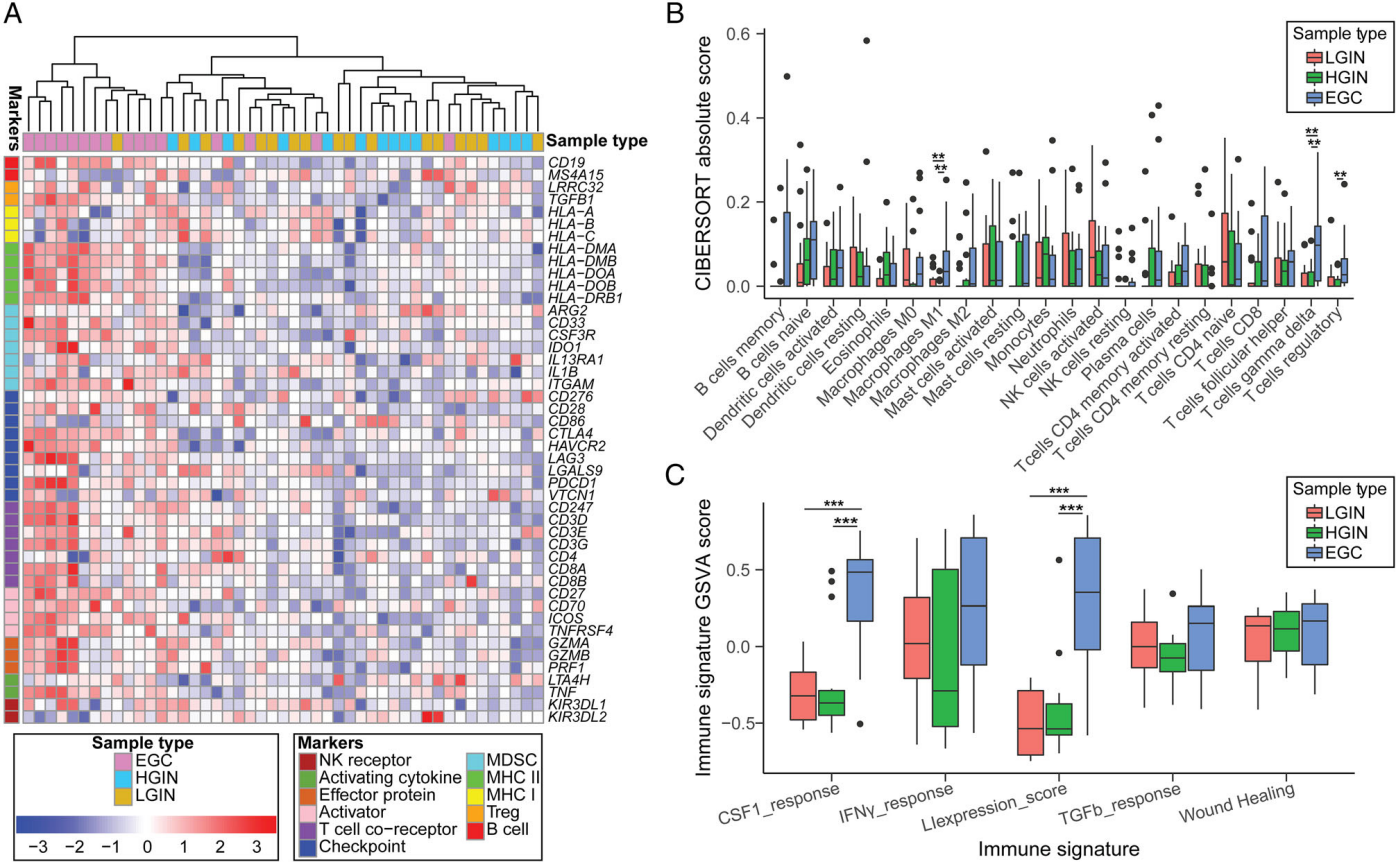
Immune microenvironment evaluation of lesions in our data. (A) Unsupervised hierarchical clustering of LGIN, HGIN, and EGC samples using 46 immune cell markers identified that EGC samples have a high score. (B) The CIBERSORT‐inferred absolute infiltration of 22 immune cell types. (C) Five immune signature scores of lesions showed a difference between EGC and precancerous lesion tissues. *p* < 0.05 was considered to be statistically significant. *p < 0.05; **p < 0.01; ***p < 0.001. Boxplots show mean ± SEM.

Based on the functional enrichment, we observed changes in immune‐related pathways in EGC. To further explore the changes in the immune microenvironment during gastric tumorigenesis, we first applied 46 immune markers of different immune cells to an unsupervised cluster of lesions and found that EGC was separated from LGIN and HGIN (Figure 4A) [26]. The infiltration scores of 22 immune cells were significantly higher in EGC than in LGIN and HGIN (*p* < 0.05), while there was no difference between LGIN and HGIN (*p* > 0.05, supplementary material, Figure S3A and Table S3) [27]. We observed that monocytes and M1 macrophages had the first and second highest median infiltration scores in EGC, both of which are related to the prognosis of many types of cancer [41, 42, 43]. We obtained the same results from the other two independent datasets (supplementary material, Figure S3B,C).

Subsequently, we used CIBERSORT [27] to estimate the infiltration of various immune cells and found that the absolute infiltration levels of gamma delta T cells and macrophages were markedly higher in EGC than in GPLs (Figure 4B and supplementary material, Figure S3D). This finding was consistent with the results estimated for five immune signatures, which showed significantly higher LIexpression_score and CSF1_response in EGC than in GPLs (Figure 4C) [28].

To further validate the relationship between gene expression and immune infiltration, we merged DEGs from TCGA STAD data with the other three DEG groups, which yielded 23 up‐regulated and five down‐regulated genes that were specifically changed in GC (EGC and TCGA STAD) (supplementary material, Figure S4A–C). We used these 28 DEGs to build a protein–protein interaction network and obtained one largest immune subnet, consisting of nine up‐regulated genes (supplementary material, Figure S4D). Using TCGA STAD to explore the relationship between these nine genes and immune infiltration, we observed that seven genes were positively correlated with the infiltration of CD8^+^ T cells and five genes were positively correlated with the infiltration of macrophages (cor > 0.3, *p* < 0.05), which was consistent with our data (supplementary material, Figure S4E–G) [34].

### Consistent DEGs play critical roles in gastric tumorigenesis and progression

As shown in supplementary material, Figure S4A,B, there were 11 up‐regulated and 11 down‐regulated coDEGs (co‐up DEGs and co‐down DEGs, supplementary material, Table S4) in all four stages (LGIN, HGIN, EGC, and TCGA STAD). All co‐up DEGs were significantly positively correlated with the stem scores, and all co‐down DEGs were significantly negatively correlated with the stem scores (|cor| > 0.35, *p* < 0.0001, supplementary material, Table S5).

**Figure 5:**
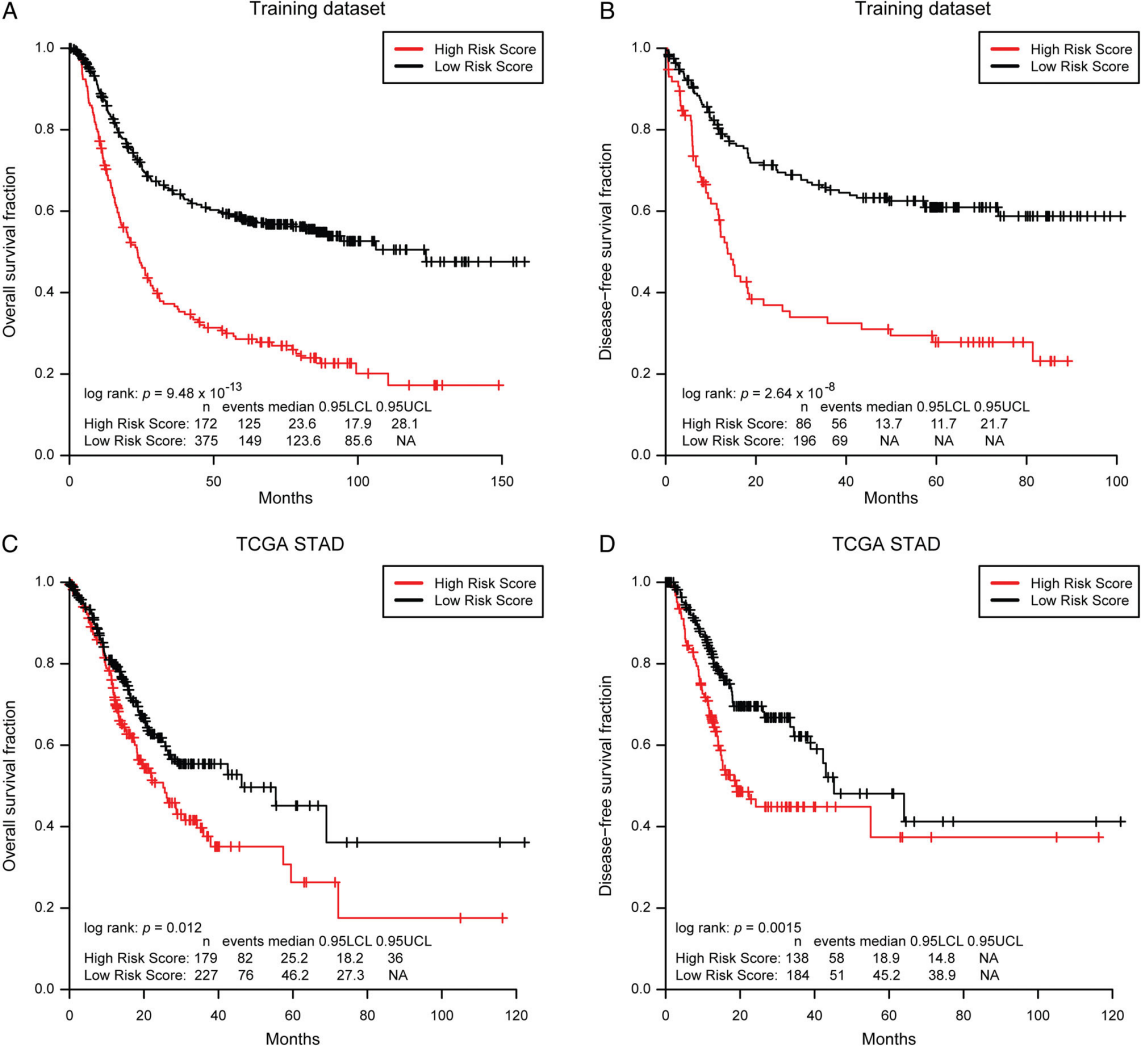
Kaplan–Meier analysis for overall survival and disease‐free survival of patients with GC according to the five‐gene signature risk score. (A, B) Kaplan–Meier analysis for (A) overall survival and (B) disease‐free survival in the training dataset (the combination of GSE62254 and GSE15460) according to the five‐gene signature risk score. (C, D) Kaplan–Meier analysis for (C) overall survival and (D) disease‐free survival in validation GC patient cohorts (TCGA STAD) according to the five‐gene signature risk score.

Given that these coDEGs continue to be dysregulated during gastric tumorigenesis and progression, we wonder what their prognostic value is. To detect the prognostic value of these 22 coDEGs, the combination (*n* = 548) of two human GC mRNA microarray datasets was designated as the training dataset. The LASSO Cox regression model was used to narrow down the 22 coDEGs to select the most useful prognostic markers, which identified five genes (*GADD45B*, *LAMP3*, *PLEKHS1*, *RGN*, and *TIMP1*).We then constructed a five‐gene signature and derived a formula to calculate the survival risk score for each patient based on the expression value of the five genes and weighted by the regression coefficient in the training dataset. We divided patients into a high‐risk group or a low‐risk group using the optimum cutoff score (cutoff = 3.79) obtained by X‐tile plot. Patients with lower risk scores had a better OS (*n* = 547, log rank: *p* = 9.48 × 10^−13^, Figure 5A) and disease‐free survival (DFS, *n* = 300, log rank: *p* = 2.64 × 10^−8^, Figure 5B) than patients with higher risk scores.

### Validation of the prognostic significance of the five‐gene signature in GC

The genes in the five‐gene signature participate in many important biological processes, including growth (*GADD45B* and *TIMP1*), metabolism (*TIMP1* and *RGN*), signal transduction (*PLEKHS1*), extracellular matrix assembly (*TIMP1*), and antigen presentation (*LAMP3*). Univariate and multivariate Cox proportional hazard regression analyses showed that the five‐gene signature was an independent hazardous prognostic factor associated with OS (HR = 1.94, *p* = 9.26 × 10^−8^) and DFS (HR = 2.27, *p* = 1.24 × 10^−5^) of GC patients from other covariates, including gender, age, and pathological stage (supplementary material, Table S6). We obtained the same results in each individual microarray dataset of the training dataset with the same formula and the same cutoff score (cutoff = 3.79) (*p* < 0.05, supplementary material, Figure S5A,B and Table S6).

To further validate the prognostic value of the five‐gene signature, we used the same formula and the same cutoff (cutoff = 3.79) to dichotomize TCGA STAD patients (independent testing dataset) into high‐ or low‐risk groups. Similar to the findings from the training set, patients in the high‐risk group had a shorter median OS (log rank: *p* = 0.012, Figure 5C) and DFS (log rank: *p* = 0.0015, Figure 5D) than patients in the low‐risk group. The univariate and multivariate Cox proportional hazard regression analyses showed that the prognostic capacities of the five‐gene signature in OS and DFS were independent of the aforementioned covariates (supplementary material, Table S6).

**Figure 6:**
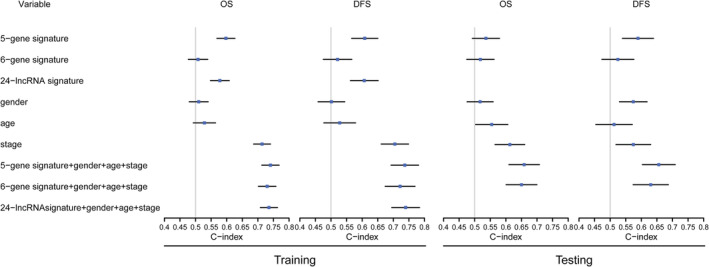
Prognostic accuracy comparison between the five‐gene signature and the other two established GC RNA expression signatures. Harrell's concordance index (C‐index) was used to assess the discrimination and prognostic accuracy of the signatures. Forest plots illustrating the C‐index (95% CI) for OS and DFS in training (left) and testing (right). CI, confidence interval.

Additionally, we compared our five‐gene signature with two established RNA expression signatures, including a six‐gene signature [13] and a 24‐lncRNA signature [44] for GC. In all the training, GSE62254, GSE15460, and TCGA STAD datasets, our five‐gene signature achieved a higher C‐index in OS or DFS than the six‐gene signature (Figure 6). Moreover, in three of the four datasets or both datasets with DFS data (GSE62254 and TCGA STAD data), the combination of the aforementioned covariates with our five‐gene signature provided a higher prognostic accuracy of OS or DFS than with the six‐gene signature (Figure 6 and supplementary material, Figure S6). Only the microarray datasets that were downloaded from the GEO database contain all 24 lncRNAs in the 24‐lncRNA signature. Similarly, for all the training, GSE62254, and GSE15460 datasets, our five‐gene signature showed a higher C‐index in OS or DFS than the 24‐lncRNA signature that was trained in the GSE62254 dataset (Figure 6 and supplementary material, Figure S6). In addition, the combination of covariates with our five‐gene signature provided a higher prognostic accuracy of OS and DFS than with the 24‐lncRNA signature (Figure 6 and supplementary material, Figure S6).

## Discussion

Over the past few decades, to eradicate tumors, scientists have focused mainly on tumors themselves. Few advances have been made in understanding the mechanisms by which GPLs become early invasive tumors and by which tumors escape attack of the immune system and therapies. In this project, we detected the expression profiles of GPL and EGC samples to investigate the mechanisms underlying gastric tumorigenesis.

First, DEG enrichment showed that gastric epithelial cells first displayed changes in proliferation and development, followed by changes in cell adhesion and gastric acid secretion; finally, the detoxication was reduced along with immune microenvironment activation during gastric tumorigenesis, which has never been integrally elaborated before. As shown in Figure 2, chromatin remodeling‐related pathways (chromatin remodeling at centromere and CENP‐A containing nucleosome assembly) and their regulated pathways (chromatin assembly or disassembly, nucleosome organization, DNA packaging, and protein–DNA complex assembly) that are frequently altered in GC [8, 45, 46, 47] were dysregulated in LGIN and HGIN. Cell adhesion‐related pathways are the top perturbed pathways in GC [47, 48, 49]. The dysregulation of the Rap1 signaling pathway and calcium‐independent cell–cell adhesion via plasma membrane cell‐adhesion molecules in HGIN indicated acquisition of the potential for invasion and metastasis of HGIN [46, 50]. Severe inflammation could promote the transition from a precancerous state to tumorigenesis [9, 51]. The single‐cell sequencing results suggested that macrophages have a critical role in promoting gastric tumorigenesis [12]. In our project, the cytokine‐ and inflammation‐related pathways were significantly up‐regulated in EGC, which might contribute to the formation of EGC.

We observed cancer‐like molecular expression profiles and phenotypes appearing in LGIN that were sustained in HGIN and EGC, such as some tumor hallmarks, stem‐cell‐like features, commonly changed driver genes, and common DEGs. Even so, the expression profiles of LGIN and HGIN were more similar to each other than to that of EGC, despite the greater similarities in morphology between HGIN and EGC. The most obvious difference between EGC and GPL was the difference in the immune microenvironment. The immune cell infiltration in EGC was significantly higher than that in the GPL, especially the infiltration of lymphocytes and macrophages. Tumor‐infiltrating lymphocytes are supposedly associated with a positive prognosis for GC patients and are considered to reflect the protective host antitumor immune response [52, 53]; however, tumor‐associated macrophages are considered to play a complicated role in GC development by contributing to the progression and poor prognosis of GC [41, 42, 54] while also indicating better OS [54].

Intriguingly, we found no significant difference in the stemness between EGC and paired controls, which might be interpreted as the influence of the tumor and the tumor microenvironment on the normal mucosa in view of the significant differences between LGIN or HGIN and their paired controls, between the EGC controls and the LGIN and HGIN controls, and the lack of significant differences among LGIN, HGIN, and EGC in their stem scores. Simultaneously, the immune infiltration was significantly higher in EGC than in GPL (especially macrophages) [12]. The influence of the tumor and tumor microenvironment serves to assimilate the surrounding normal mucosa, which explained to some degree why patients who underwent gastric endoscopic submucosal dissection (ESD) had a higher annual incidence of recurrent GC than patients who underwent surgery [55].

According to the central dogma of genetics, RNA is the next‐level executor of DNA. In the past, however, scientists have primarily focused on driver gene mutations and found that intestinal‐type GC shows no specific mutation patterns during tumorigenesis [7]. We checked the expression profiles of 299 pan‐cancer‐related driver genes [56] in gastric tumorigenesis and found consistent differentially expressed driver genes in GPLs and EGC, including *BCL2L11*, *RET*, *ALB*, *GRIN2D*, and *BRCA1*. *BCL2L11*, which has been considered to act as an apoptotic activator, showed a significant decrease in gastric lesions [57]. *GRIN2D*, which has been deemed to be a possible oncogene in pan‐cancer, showed a significant increase in gastric lesions [56, 58]. These two driver genes might play vital roles in gastric tumorigenesis, and other variational driver genes might change to sustain the balance of biological functions, such as *BRCA1*, which has been identified as a tumor suppressor and showed increased expression in gastric lesions; and *TP53*, which has also been identified as a tumor suppressor and showed increased expression in LGIN tissues. Moreover, these coincidentally changed driver genes showed significant correlations with dedifferentiation, indicating that expression of these driver genes plays equally critical roles in functional maintenance.

In this study, we identified 22 coDEGs that might participate in the dedifferentiation of gastric epithelium cells, with the co‐up DEGs being positively correlated with the stem score and the co‐down DEGs being negatively correlated with the stem score. In addition, we calculated the prognostic relevance of these coDEGs and developed a robust five‐gene signature. The five‐gene signature could predict OS and DFS and was an independent prognostic factor associated with OS and DFS. The prognostic robustness was validated in an independent cohort and the discrimination and prognostic accuracy were assessed and compared with two established signatures for GC.

To the best of our knowledge, this study is the first to include paired continuous lesions representing gastric tumorigenesis. Our findings showed that during gastric tumorigenesis, cancer‐like changes occur in LGIN and accumulate in HGIN and EGC. EGC has a more active immune microenvironment than GPLs. EGC tissues, but not GPL tissues, could assimilate the gene expression of the surrounding normal mucosa. The five‐gene signature identified from the tumorigenesis process showed robust prognostic significance for OS and DFS in GC patients, and might lead to the generation of potential molecular targets for the development of anticancer therapy.

## Author contributions statement

SJC and AMY conceived and designed the project. CLZ performed the experiments. YJZ analyzed the data and wrote the manuscript. XW, JQW, GJF, and XBD collected the clinical samples and conducted the follow‐up. XW, LF, and XHL revised the manuscript.

## Data availability statement

The raw data and processed data have been deposited in the National Center for Biotechnology Information (NCBI) Gene Expression Omnibus (GEO) database and are accessible through GEO Series accession number GES130823 (https://www.ncbi.nlm.nih.gov/geo/query/acc.cgi?acc=GSE130823).

## Supporting information


**Supplementary materials and methods**

**Supplementary figure legends**

**Figure S1.** Venn diagrams illustrating candidate DEGs in different group samples

**Figure S2.** Correlation of stem scores and two consistent changed driver genes in LGIN, HGIN, and EGC

**Figure S3.** Immune microenvironment evaluation of lesions

**Figure S4.** Correlation between representative gene expression and immune infiltration of TCGA STAD data

**Figure S5.** Kaplan–Meier analysis for overall survival of patients with GC according to the five‐gene signature risk score

**Figure S6.** Prognostic accuracy comparison between the five‐gene signature and two other established GC RNA expression signatures in GSE62254 and GSE15460

**Table S1.** Clinical information for each individual
**Table S2.** Sample information of the mRNA microarray
**Table S3.** Mann–Whitney test of immune infiltration scores
**Table S4.** Twenty‐two coDEGs from merging four groups of DEGs
**Table S5.** Correlations between DEGs and stem scores
**Table S6.** Univariate and multivariate Cox proportional hazard regression analysis of overall survival and disease‐free survival in training (*n* = 547), testing (*n* = 406), GSE62254 (*n* = 300), and GSE15460 (*n* = 248) patients according to the five‐gene signature, gender, age, and stage

## References

[1] Bray F , Ferlay J , Soerjomataram I , *et al* Global cancer statistics 2018: GLOBOCAN estimates of incidence and mortality worldwide for 36 cancers in 185 countries. CA Cancer J Clin 2018; 68 **:** 394–424. 30207593 10.3322/caac.21492

[2] Tan P , Yeoh KG . Genetics and molecular pathogenesis of gastric adenocarcinoma. Gastroenterology 2015; 149 **:** 1153–1162.e3. 26073375 10.1053/j.gastro.2015.05.059

[3] Van Cutsem E , Sagaert X , Topal B , *et al* Gastric cancer. Lancet 2016; 388 **:** 2654–2664. 27156933 10.1016/S0140-6736(16)30354-3

[4] Correa P . A human model of gastric carcinogenesis. Cancer Res 1988; 48 **:** 3554–3560. 3288329

[5] El‐Rifai W , Frierson HF Jr , Harper JC , *et al* Expression profiling of gastric adenocarcinoma using cDNA array. Int J Cancer 2001; 92 **:** 832–838. 11351303 10.1002/ijc.1264

[6] Hippo Y , Taniguchi H , Tsutsumi S , *et al* Global gene expression analysis of gastric cancer by oligonucleotide microarrays. Cancer Res 2002; 62 **:** 233–240. 11782383

[7] Maesawa C , Tamura G , Suzuki Y , *et al* The sequential accumulation of genetic alterations characteristic of the colorectal adenoma–carcinoma sequence does not occur between gastric adenoma and adenocarcinoma. J Pathol 1995; 176 **:** 249–258. 7674088 10.1002/path.1711760307

[8] Boussioutas A , Li H , Liu J , *et al* Distinctive patterns of gene expression in premalignant gastric mucosa and gastric cancer. Cancer Res 2003; 63 **:** 2569–2577. 12750281

[9] Valente P , Garrido M , Gullo I , *et al* Epithelial dysplasia of the stomach with gastric immunophenotype shows features of biological aggressiveness. Gastric Cancer 2015; 18 **:** 720–728. 25146833 10.1007/s10120-014-0416-5

[10] Lee HJ , Nam KT , Park HS , *et al* Gene expression profiling of metaplastic lineages identifies CDH17 as a prognostic marker in early stage gastric cancer. Gastroenterology 2010; 139 **:** 213–225.e3. 20398667 10.1053/j.gastro.2010.04.008 PMC2917327

[11] Li D , Bautista MC , Jiang SF , *et al* Risks and predictors of gastric adenocarcinoma in patients with gastric intestinal metaplasia and dysplasia: a population‐based study. Am J Gastroenterol 2016; 111 **:** 1104–1113. 27185078 10.1038/ajg.2016.188

[12] Zhang P , Yang M , Zhang Y , *et al* Dissecting the single‐cell transcriptome network underlying gastric premalignant lesions and early gastric cancer. Cell Rep 2019; 27 **:** 1934–1947.e5. 31067475 10.1016/j.celrep.2019.04.052

[13] Cho JY , Lim JY , Cheong JH , *et al* Gene expression signature‐based prognostic risk score in gastric cancer. Clin Cancer Res 2011; 17 **:** 1850–1857. 21447720 10.1158/1078-0432.CCR-10-2180 PMC3078023

[14] Takeno A , Takemasa I , Seno S , *et al* Gene expression profile prospectively predicts peritoneal relapse after curative surgery of gastric cancer. Ann Surg Oncol 2010; 17 **:** 1033–1042. 20012501 10.1245/s10434-009-0854-1

[15] Chen CN , Lin JJ , Chen JJ , *et al* Gene expression profile predicts patient survival of gastric cancer after surgical resection. J Clin Oncol 2005; 23 **:** 7286–7295. 16145069 10.1200/JCO.2004.00.2253

[16] Lee J , Sohn I , Do IG , *et al* Nanostring‐based multigene assay to predict recurrence for gastric cancer patients after surgery. PLoS One 2014; 9 **:** e90133. 24598828 10.1371/journal.pone.0090133 PMC3943911

[17] Bosman FT , Carneiro F , Hruban RH , *et al* WHO Classification of Tumours of the Digestive System (Vol. 4). IARC Press: Lyon, 2010.

[18] Dixon MF , Genta RM , Yardley JH , *et al* Classification and grading of gastritis. The updated Sydney System. International Workshop on the Histopathology of Gastritis, Houston 1994. Am J Surg Pathol 1996; 20 **:** 1161–1181. 8827022 10.1097/00000478-199610000-00001

[19] World Medical Association . World Medical Association Declaration of Helsinki: ethical principles for medical research involving human subjects. JAMA 2013; 310 **:** 2191–2194. 24141714 10.1001/jama.2013.281053

[20] Kasturi SP , Skountzou I , Albrecht RA , *et al* Programming the magnitude and persistence of antibody responses with innate immunity. Nature 2011; 470 **:** 543–547. 21350488 10.1038/nature09737 PMC3057367

[21] Tamborero D , Rubio‐Perez C , Muinos F , *et al* A pan‐cancer landscape of interactions between solid tumors and infiltrating immune cell populations. Clin Cancer Res 2018; 24 **:** 3717–3728. 29666300 10.1158/1078-0432.CCR-17-3509

[22] Hanahan D , Weinberg RA . Hallmarks of cancer: the next generation. Cell 2011; 144 **:** 646–674. 21376230 10.1016/j.cell.2011.02.013

[23] Plaisier CL , Pan M , Baliga NS . A miRNA‐regulatory network explains how dysregulated miRNAs perturb oncogenic processes across diverse cancers. Genome Res 2012; 22 **:** 2302–2314. 22745231 10.1101/gr.133991.111 PMC3483559

[24] Hanzelmann S , Castelo R , Guinney J . GSVA: gene set variation analysis for microarray and RNA‐seq data. BMC Bioinf 2013; 14 **:** 7. 10.1186/1471-2105-14-7 PMC3618321 23323831

[25] Malta TM , Sokolov A , Gentles AJ , *et al* Machine learning identifies stemness features associated with oncogenic dedifferentiation. Cell 2018; 173 **:** 338–354.e15. 29625051 10.1016/j.cell.2018.03.034 PMC5902191

[26] Cancer Genome Atlas Research Network . Comprehensive and integrative genomic characterization of hepatocellular carcinoma. Cell 2017; 169 **:** 1327–1341.e23. 28622513 10.1016/j.cell.2017.05.046 PMC5680778

[27] Chen B , Khodadoust MS , Liu CL , *et al* Profiling tumor infiltrating immune cells with CIBERSORT. Methods Mol Biol 1711; 2018 **:** 243–259. 10.1007/978-1-4939-7493-1_12 PMC5895181 29344893

[28] Thorsson V , Gibbs DL , Brown SD , *et al* The immune landscape of cancer. Immunity 2018; 48 **:** 812–830.e14. 29628290 10.1016/j.immuni.2018.03.023 PMC5982584

[29] Beck AH , Espinosa I , Edris B , *et al* The macrophage colony‐stimulating factor 1 response signature in breast carcinoma. Clin Cancer Res 2009; 15 **:** 778–787. 19188147 10.1158/1078-0432.CCR-08-1283 PMC2987696

[30] Wolf DM , Lenburg ME , Yau C , *et al* Gene co‐expression modules as clinically relevant hallmarks of breast cancer diversity. PLoS One 2014; 9 **:** e88309. 24516633 10.1371/journal.pone.0088309 PMC3917875

[31] Calabro A , Beissbarth T , Kuner R , *et al* Effects of infiltrating lymphocytes and estrogen receptor on gene expression and prognosis in breast cancer. Breast Cancer Res Treat 2009; 116 **:** 69–77. 18592372 10.1007/s10549-008-0105-3

[32] Teschendorff AE , Gomez S , Arenas A , *et al* Improved prognostic classification of breast cancer defined by antagonistic activation patterns of immune response pathway modules. BMC Cancer 2010; 10 **:** 604. 21050467 10.1186/1471-2407-10-604 PMC2991308

[33] Chang HY , Sneddon JB , Alizadeh AA , *et al* Gene expression signature of fibroblast serum response predicts human cancer progression: similarities between tumors and wounds. PLoS Biol 2004; 2 **:** E7. 14737219 10.1371/journal.pbio.0020007 PMC314300

[34] Li T , Fan J , Wang B , *et al* TIMER: a web server for comprehensive analysis of tumor‐infiltrating immune cells. Cancer Res 2017; 77 **:** e108–e110. 29092952 10.1158/0008-5472.CAN-17-0307 PMC6042652

[35] Qu L , Wang ZL , Chen Q , *et al* Prognostic value of a long non‐coding RNA signature in localized clear cell renal cell carcinoma. Eur Urol 2018; 74 **:** 756–763. 30143382 10.1016/j.eururo.2018.07.032

[36] Zhang JX , Song W , Chen ZH , *et al* Prognostic and predictive value of a microRNA signature in stage II colon cancer: a microRNA expression analysis. Lancet Oncol 2013; 14 **:** 1295–1306. 24239208 10.1016/S1470-2045(13)70491-1

[37] Camp RL , Dolled‐Filhart M , Rimm DL . X‐tile: a new bio‐informatics tool for biomarker assessment and outcome‐based cut‐point optimization. Clin Cancer Res 2004; 10 **:** 7252–7259. 15534099 10.1158/1078-0432.CCR-04-0713

[38] Liu H , Kho AT , Kohane IS , *et al* Predicting survival within the lung cancer histopathological hierarchy using a multi‐scale genomic model of development. PLoS Med 2006; 3 **:** e232. 16800721 10.1371/journal.pmed.0030232 PMC1483910

[39] Harrell FE Jr , Lee KL , Mark DB . Multivariable prognostic models: issues in developing models, evaluating assumptions and adequacy, and measuring and reducing errors. Stat Med 1996; 15 **:** 361–387. 8668867 10.1002/(SICI)1097-0258(19960229)15:4<361::AID-SIM168>3.0.CO;2-4

[40] Nardone G , Rocco A , Malfertheiner P . *Helicobacter pylori* and molecular events in precancerous gastric lesions. Aliment Pharmacol Ther 2004; 20 **:** 261–270. 15274662 10.1111/j.1365-2036.2004.02075.x

[41] Peng LS , Zhang JY , Teng YS , *et al* Tumor‐associated monocytes/macrophages impair NK‐cell function via TGFβ1 in human gastric cancer. Cancer Immunol Res 2017; 5 **:** 248–256. 28148545 10.1158/2326-6066.CIR-16-0152

[42] Yamaguchi T , Fushida S , Yamamoto Y , *et al* Tumor‐associated macrophages of the M2 phenotype contribute to progression in gastric cancer with peritoneal dissemination. Gastric Cancer 2016; 19 **:** 1052–1065. 26621525 10.1007/s10120-015-0579-8 PMC5034006

[43] Nie Y , Chen J , Huang D , *et al* Tumor‐associated macrophages promote malignant progression of breast phyllodes tumors by inducing myofibroblast differentiation. Cancer Res 2017; 77 **:** 3605–3618. 28512246 10.1158/0008-5472.CAN-16-2709

[44] Zhu X , Tian X , Yu C , *et al* A long non‐coding RNA signature to improve prognosis prediction of gastric cancer. Mol Cancer 2016; 15 **:** 60. 27647437 10.1186/s12943-016-0544-0 PMC5029104

[45] Takeshima H , Niwa T , Takahashi T , *et al* Frequent involvement of chromatin remodeler alterations in gastric field cancerization. Cancer Lett 2015; 357 **:** 328–338. 25462860 10.1016/j.canlet.2014.11.038

[46] Zang ZJ , Cutcutache I , Poon SL , *et al* Exome sequencing of gastric adenocarcinoma identifies recurrent somatic mutations in cell adhesion and chromatin remodeling genes. Nat Genet 2012; 44 **:** 570–574. 22484628 10.1038/ng.2246

[47] Wang K , Kan J , Yuen ST , *et al* Exome sequencing identifies frequent mutation of *ARID1A* in molecular subtypes of gastric cancer. Nat Genet 2011; 43 **:** 1219–1223. 22037554 10.1038/ng.982

[48] Wang K , Yuen ST , Xu J , *et al* Whole‐genome sequencing and comprehensive molecular profiling identify new driver mutations in gastric cancer. Nat Genet 2014; 46 **:** 573–582. 24816253 10.1038/ng.2983

[49] Cancer Genome Atlas Research Network . Comprehensive molecular characterization of gastric adenocarcinoma. Nature 2014; 513 **:** 202–209. 25079317 10.1038/nature13480 PMC4170219

[50] Cavallaro U , Christofori G . Cell adhesion and signalling by cadherins and Ig‐CAMs in cancer. Nat Rev Cancer 2004; 4 **:** 118–132. 14964308 10.1038/nrc1276

[51] Guo Y , Nie Q , MacLean AL , *et al* Multiscale modeling of inflammation‐induced tumorigenesis reveals competing oncogenic and oncoprotective roles for inflammation. Cancer Res 2017; 77 **:** 6429–6441. 28951462 10.1158/0008-5472.CAN-17-1662

[52] Zhang D , He W , Wu C , *et al* Scoring system for tumor‐infiltrating lymphocytes and its prognostic value for gastric cancer. Front Immunol 2019; 10 **:** 71. 30761139 10.3389/fimmu.2019.00071 PMC6361780

[53] Kang BW , Seo AN , Yoon S , *et al* Prognostic value of tumor‐infiltrating lymphocytes in Epstein–Barr virus‐associated gastric cancer. Ann Oncol 2016; 27 **:** 494–501. 26673353 10.1093/annonc/mdv610

[54] Zhang H , Wang X , Shen Z , *et al* Infiltration of diametrically polarized macrophages predicts overall survival of patients with gastric cancer after surgical resection. Gastric Cancer 2015; 18 **:** 740–750. 25231913 10.1007/s10120-014-0422-7

[55] Hahn KY , Park CH , Lee YK , *et al* Comparative study between endoscopic submucosal dissection and surgery in patients with early gastric cancer. Surg Endosc 2018; 32 **:** 73–86. 28639042 10.1007/s00464-017-5640-8

[56] Bailey MH , Tokheim C , Porta‐Pardo E , *et al* Comprehensive characterization of cancer driver genes and mutations. Cell 2018; 173 **:** 371–385 e18. 29625053 10.1016/j.cell.2018.02.060 PMC6029450

[57] O'Connor L , Strasser A , O'Reilly LA , *et al* Bim: a novel member of the Bcl‐2 family that promotes apoptosis. EMBO J 1998; 17 **:** 384–395. 9430630 10.1093/emboj/17.2.384 PMC1170389

[58] Sharma A , Jiang C , De S . Dissecting the sources of gene expression variation in a pan‐cancer analysis identifies novel regulatory mutations. Nucleic Acids Res 2018; 46 **:** 4370–4381. 29672706 10.1093/nar/gky271 PMC5961375

