# Supplementary material for: Dissecting expression profiles of gastric precancerous lesions and early gastric cancer to explore crucial molecules in intestinal‐type gastric cancer tumorigenesis
Source: J Pathol. 2020 May 27;251(2):135–46. doi: 10.1002/path.5434 (PMC7317417; doi:10.1002/path.5434)
Supplement: Supplementary file 1 — Supplementary materials and methods [file PATH-251-135-s001.docx]

**Dissecting expression profiles of gastric precancerous lesions and early gastric cancer to explore crucial molecules in intestinal-type gastric cancer tumorigenesis**

Zhang *et al. J Pathol* DOI: 10.1002/path.5434

**Supplementary materials and methods**

Reference numbers refer to the main text list

**Qualification of RNA used in microarray expression profiling**

RNA concentrations were determined using a NanoDrop ND-2000 Spectrophotometer (NanoDrop Technologies, Wilmington, DE, USA), and RNA integrity was evaluated using a 2100 Bioanalyzer (Agilent Technologies, Santa Clara, CA, USA). RNA samples with a concentration > 40 ng/µl, 1.8 < OD_260/280_ < 2.0, and integrity number ≥ 6 were used in the microarray analysis.

**Data processing for the downloaded microarray data**

The expression values of probes were retrieved via a robust multi-array average (RMA) algorithm and further quantile-normalized using the Bioconductor package ‘affy’. The ComBat algorithm was utilized to eliminate potential batch effects. The expression value for a particular gene that was mapped by multiple probes was determined as the probe with the highest median expression value across all samples, as described in the main text.

**Stem scores production**

To calculate the stemness indices, the stemness signature was firstly identified and validated by machine learning. The one-class logistic regression (OCLR) was used on the pluripotent stem cell samples (ESC and iPSC) from the Progenitor Cell Biology Consortium (PCBC) dataset to construct a predictive signature, and leave-one-out cross-validation was applied to test the accuracy of the signature. Then the Spearman correlations between the signature’s weight vector and the expression profile of the samples were computed and mapped to the [0,1] range by using a linear transformation that subtracted the minimum and divided by the maximum, which obtained the final stem scores. The stemness index workflow can be found at the following link: https://bioinformaticsfmrp.github.io/PanCanStem_Web/ [24].
