# Supplementary material for: Dissecting expression profiles of gastric precancerous lesions and early gastric cancer to explore crucial molecules in intestinal‐type gastric cancer tumorigenesis
Source: J Pathol. 2020 May 27;251(2):135–46. doi: 10.1002/path.5434 (PMC7317417; doi:10.1002/path.5434)
Supplement: Supplementary file 2 — Supplementary figure legends [file PATH-251-135-s002.doc]

**Dissecting expression profiles of gastric precancerous lesions and early gastric cancer to explore crucial molecules in intestinal-type gastric cancer tumorigenesis**

Zhang *et al. J Pathol* DOI: 10.1002/path.5434

**Supplementary figure legends**

**Figure S1. Venn diagrams illustrating candidate DEGs in different group samples.** (A) Up-regulated and (B) down-regulated DEGs in paired LGIN, HGIN, and EGC group samples, respectively. (C) Up-regulated, (D) down-regulated, and (E) all dysregulated DEGs in EGC relative to LGIN or HGIN, respectively.

**Figure S2. Correlation of stem scores and two consistent changed driver genes in LGIN, HGIN, and EGC.** *p*< 0.05 was considered to be statistically significant.

**Figure S3. Immune microenvironment evaluation of lesions.** (A–C) GSVA scores of 22 types of infiltrated immune cells in (A) our data and (B, C) two independent cohorts. All three cohorts show that EGC samples have a high infiltration score. (D) The CIBERSORT-inferred relative fractions of 22 types of infiltrated immune cells in our samples.

**Figure S4. Correlation between representative gene expression and immune infiltration of TCGA STAD data.** (A) Up-regulated and (B) down-regulated DEGs in LGIN, HGIN, EGC, and TCGA STAD group samples. STAD_TCGA represents TCGA STAD data. (C) GC-specific DEGs and (D) the largest protein–protein interaction network (PPI) constructed by these DEGs. (E–G) Correlation between expression of PPI-related genes and tumor purity, infiltration of CD8^+^ T cells or macrophages. *p*< 0.05 was considered to be statistically significant.

**Figure S5. Kaplan–Meier analysis for overall survival of patients with GC according to the five-gene signature risk score.** (A, B) Kaplan–Meier analysis for overall survival in (A) GSE15460 and (B) GSE62254 according to the five-gene signature risk scores.

**Figure S6. Prognostic accuracy comparison between the five-gene signature and two other established GC RNA expression signatures in GSE62254 and GSE15460.** Forest plots illustrating the C-index (95% CI) for OS and DFS in GSE62254 and GSE15460. CI, confidence interval.
