# Supplementary figures and images for: Dissecting expression profiles of gastric precancerous lesions and early gastric cancer to explore crucial molecules in intestinal‐type gastric cancer tumorigenesis
Source: J Pathol. 2020 May 27;251(2):135–46. doi: 10.1002/path.5434 (PMC7317417; doi:10.1002/path.5434)

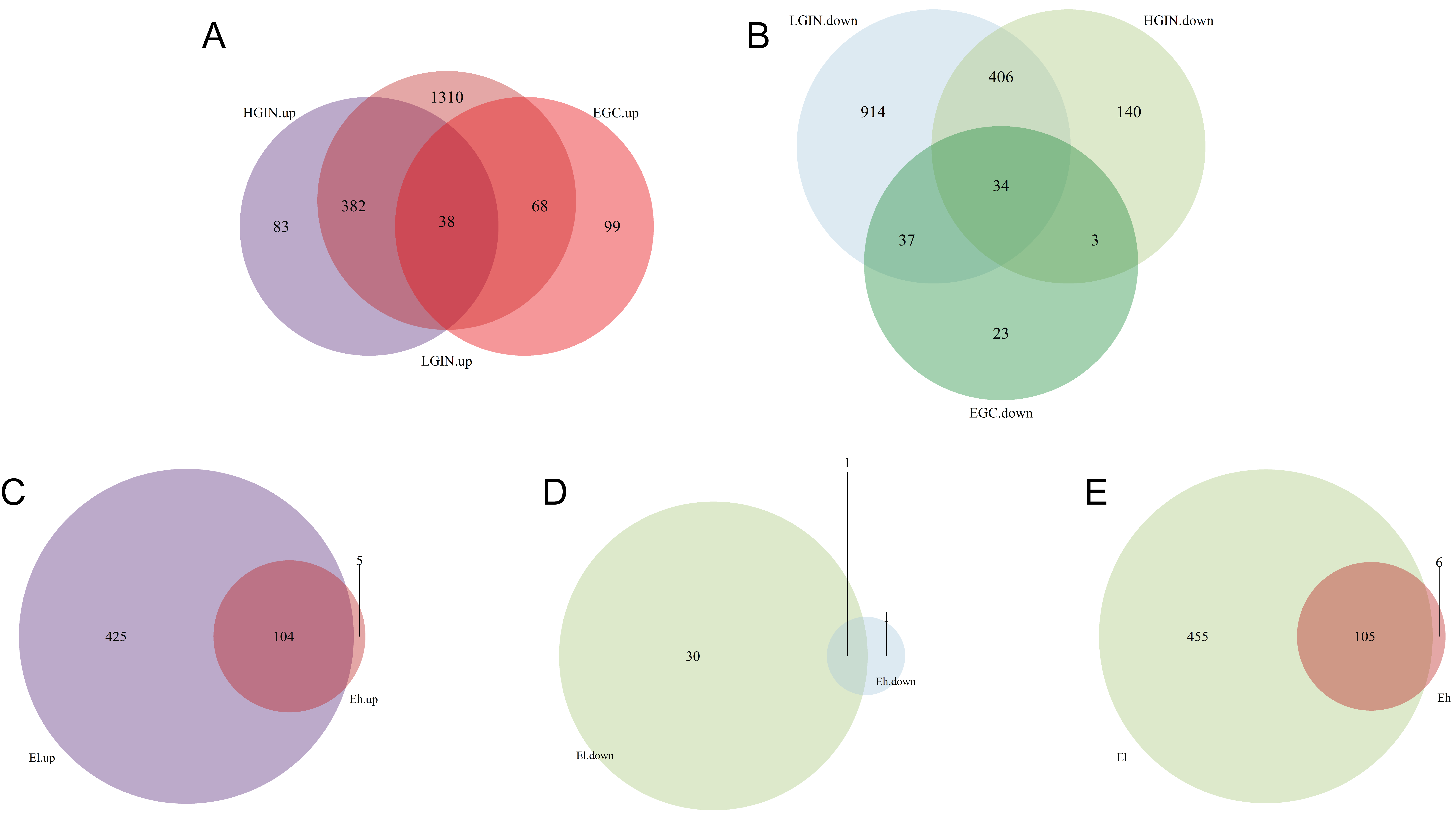

Supplement: Supplementary file 3 — Figure S1. Venn diagrams illustrating candidate DEGs in different group samples [file PATH-251-135-s003.tif]

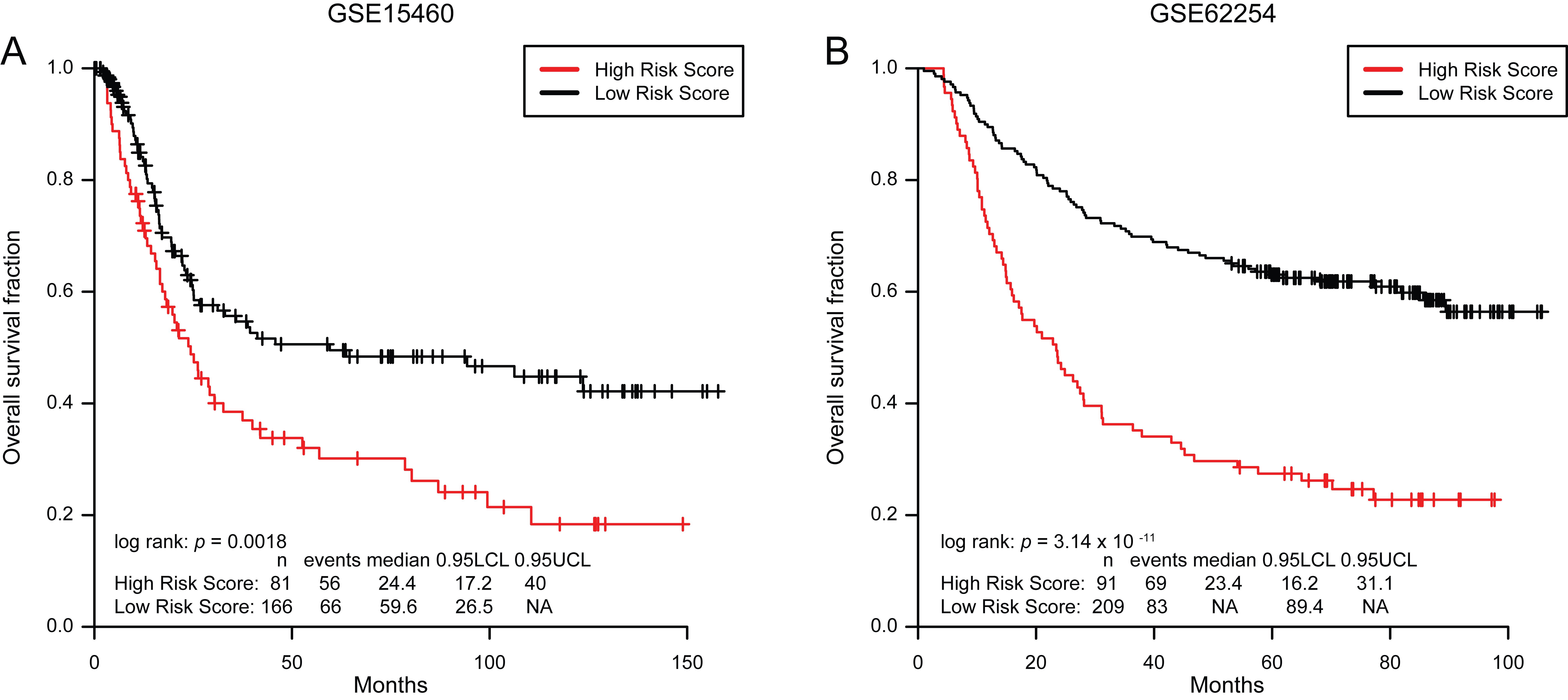

Supplement: Supplementary file 5 — Figure S3. Immune microenvironment evaluation of lesions [file PATH-251-135-s005.tif]

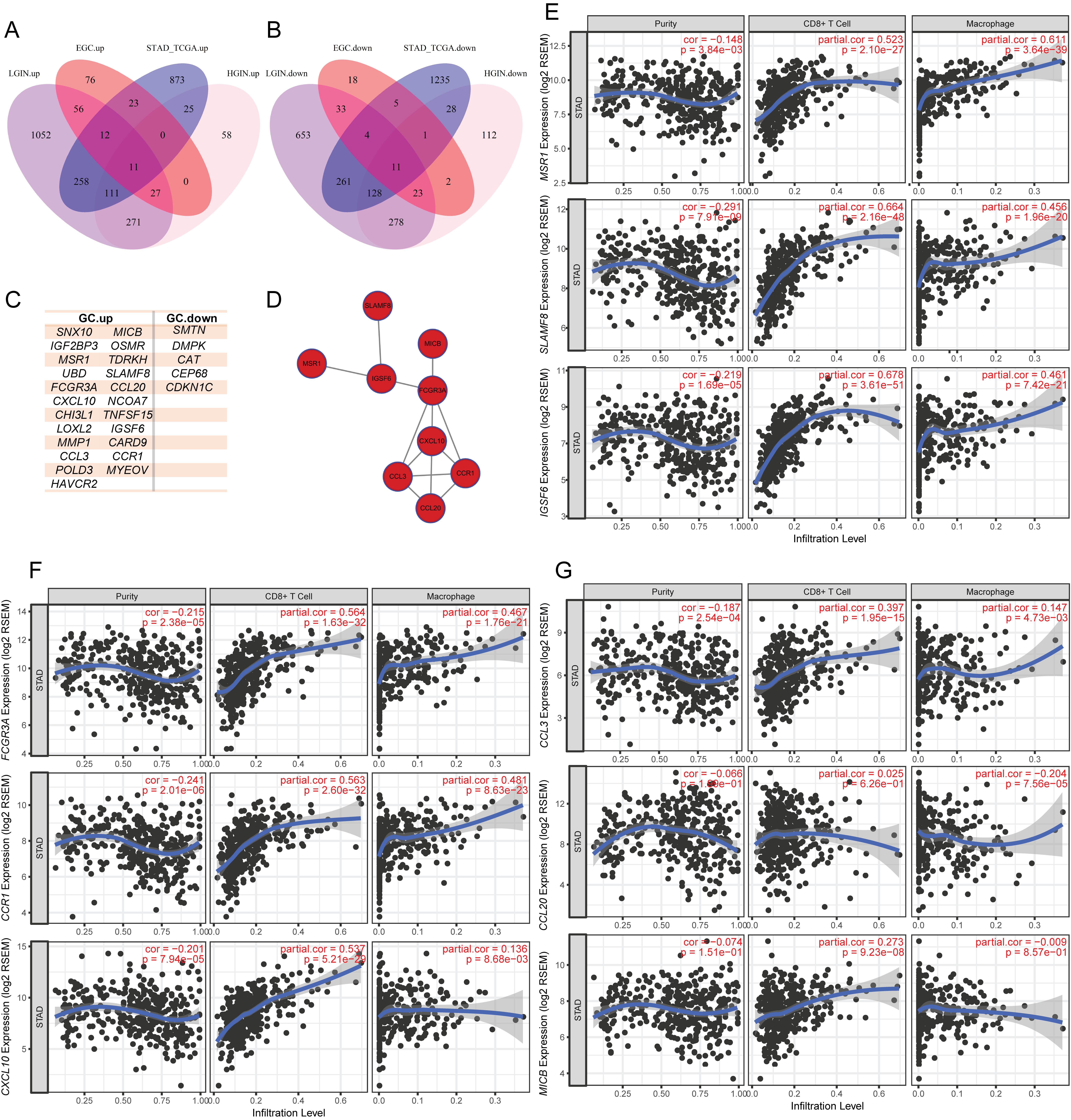

Supplement: Supplementary file 6 — Figure S4. Correlation between representative gene expression and immune infiltration of TCGA STAD data [file PATH-251-135-s006.tif]

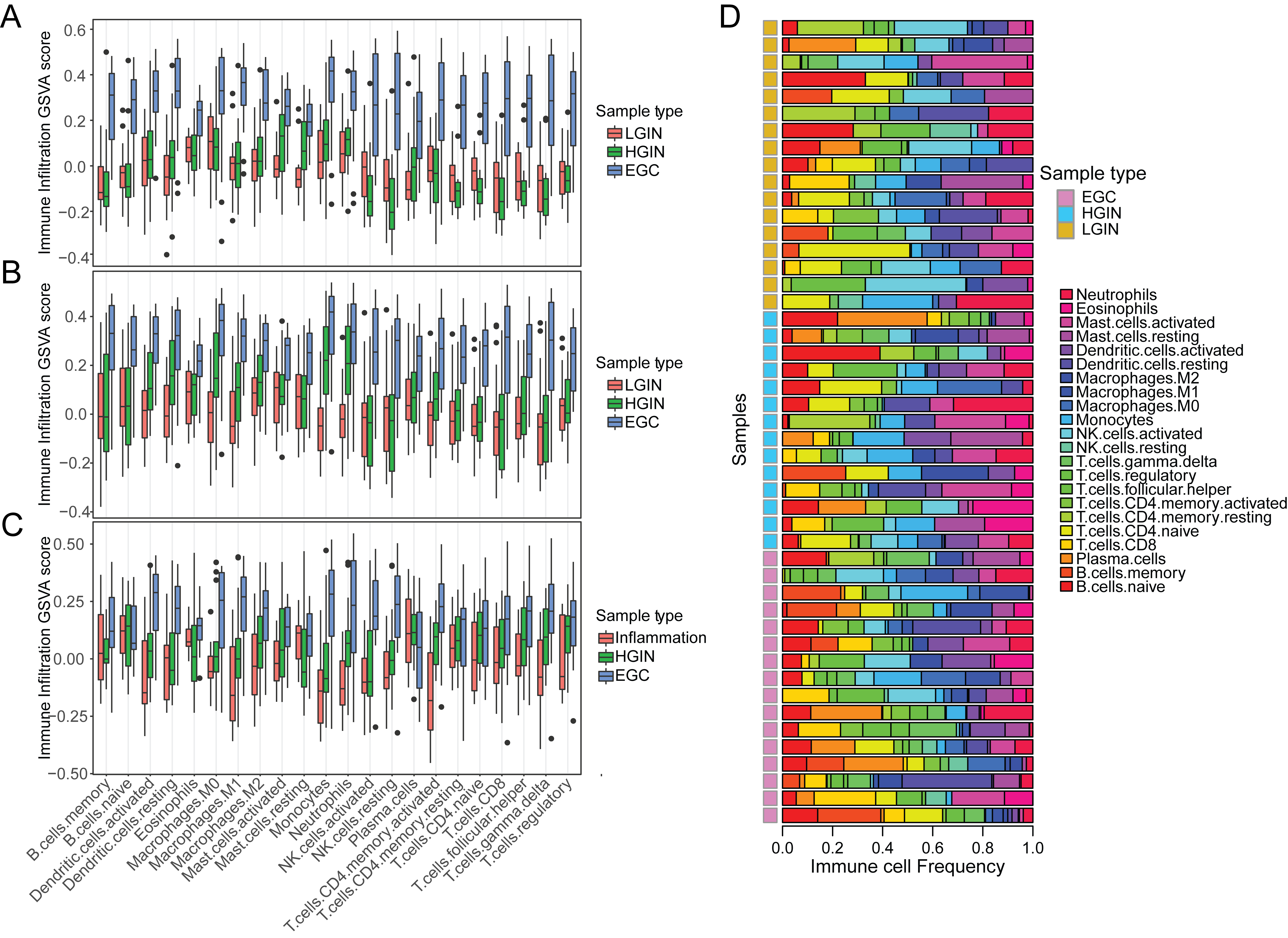

Supplement: Supplementary file 7 — Figure S5. Kaplan–Meier analysis for overall survival of patients with GC according to the five‐gene signature risk score [file PATH-251-135-s007.tif]

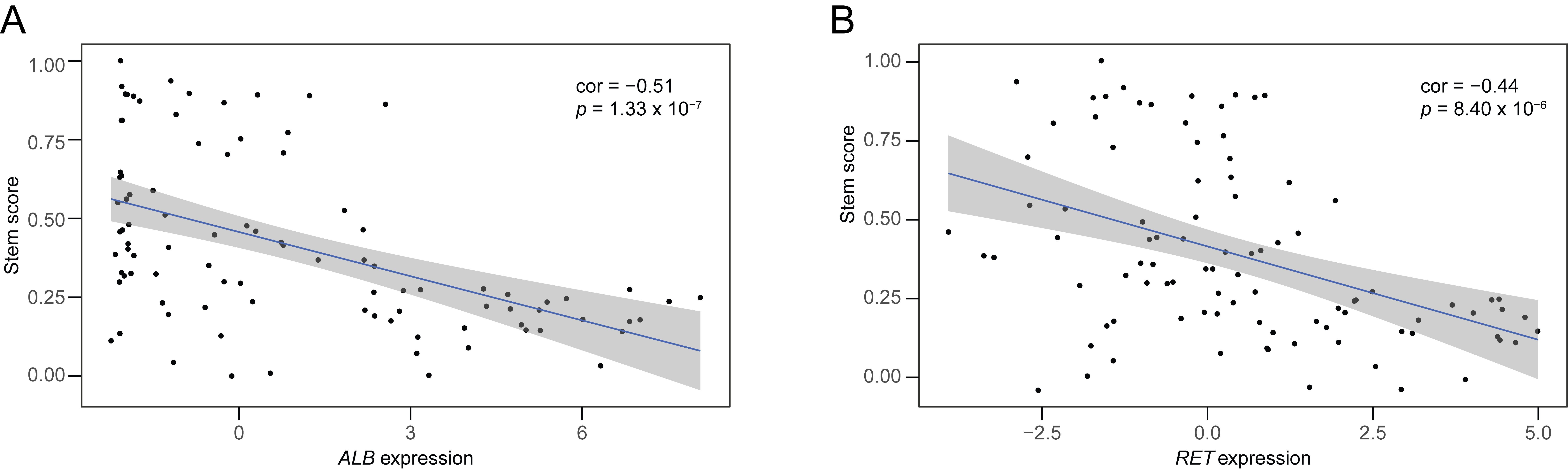

Supplement: Supplementary file 8 — Figure S6. Prognostic accuracy comparison between the five‐gene signature and two other established GC RNA expression signatures in GSE62254 and GSE15460 [file PATH-251-135-s008.tif]
