# Supplementary material for: Dissecting expression profiles of gastric precancerous lesions and early gastric cancer to explore crucial molecules in intestinal‐type gastric cancer tumorigenesis
Source: J Pathol. 2020 May 27;251(2):135–46. doi: 10.1002/path.5434 (PMC7317417; doi:10.1002/path.5434)
Supplement: Supplementary file 9 — Table S1. Clinical information for each individual Table S2. Sample information of the mRNA microarray Table S3. Mann–Whitney test of immune infiltration scores Table S4. Twenty‐two coDEGs from merging four groups of DEGs Table S5. Correlations between DEGs and stem scores Table S6. Univariate and multivariate Cox proportional hazard regression analysis of overall survival and disease‐free survival in training (n = 547), testing (n = 406), GSE62254 (n = 300), and GSE15460 (n = 248) patients according to the five‐gene signature, gender, age, and stage [file PATH-251-135-s009.doc]

**Dissecting expression profiles of gastric precancerous lesions and early gastric cancer to explore crucial molecules in intestinal-type gastric cancer tumorigenesis**

Zhang *et al. J Pathol* DOI: 10.1002/path.5434

**Supplementary tables S1–S6**

| **Table S1. Clinical information for each individual** | | | | | | |
| --- | --- | --- | --- | --- | --- | --- |
| **Patient ID** | **Age** | **Gender** | **HP status** | | **Biopsy site** | **Pathological diagnosis** |
| LGIN_1 | 74 | Female | Negative | Angular incisure | | Inflammation |
|  |  |  |  | Pyloric antrum | | LGIN |
| LGIN_2 | 61 | Male | Negative | Corpus | | Inflammation |
|  |  |  |  | Pyloric antrum | | LGIN |
| LGIN_3 | 54 | Male | Negative | Corpus | | Inflammation |
|  |  |  |  | Pyloric antrum | | LGIN |
| LGIN_4 | 60 | Female | Positive | Corpus | | Inflammation |
|  |  |  |  | Angular incisure | | LGIN |
| LGIN_5 | 61 | Female | Negative | Corpus | | Inflammation |
|  |  |  |  | Pyloric antrum | | LGIN |
| LGIN_6 | 63 | Male | Negative | Corpus | | Inflammation |
|  |  |  |  | Pyloric antrum | | LGIN |
| LGIN_7 | 60 | Female | Negative | Corpus | | Inflammation |
|  |  |  |  | Pyloric antrum | | LGIN |
| LGIN_8 | 58 | Female | Negative | Corpus | | Inflammation |
|  |  |  |  | Pyloric antrum | | LGIN |
| LGIN_9 | 58 | Female | Negative | Corpus | | Inflammation |
|  |  |  |  | Angular incisure | | LGIN |
| LGIN_10 | 44 | Male | Negative | Corpus | | Inflammation |
|  |  |  |  | Pyloric antrum | | LGIN |
| LGIN_11 | 63 | Female | Positive | Corpus | | Inflammation |
|  |  |  |  | Pyloric antrum | | LGIN |
| LGIN_12 | 58 | Female | Negative | Corpus | | Inflammation |
|  |  |  |  | Pyloric antrum | | LGIN |
| LGIN_13 | 56 | Female | Negative | Corpus | | Inflammation |
|  |  |  |  | Pyloric antrum | | LGIN |
| LGIN_14 | 59 | Female | Negative | Corpus | | Inflammation |
|  |  |  |  | Angular incisure | | LGIN |
| LGIN_15 | 55 | Female | Positive | Corpus | | Inflammation |
|  |  |  |  | Pyloric antrum | | LGIN |
| LGIN_16 | 63 | Male | Negative | Pyloric antrum | | Inflammation |
|  |  |  |  | Pyloric antrum | | LGIN |
| LGIN_17 | 56 | Male | Negative | Corpus | | Inflammation |
|  |  |  |  | Pyloric antrum | | LGIN |
| HGIN_1 | 46 | Male | Positive | Fundus | | Inflammation |
|  |  |  |  | Pyloric antrum | | HGIN |
| HGIN_2 | 56 | Male | Negative | Corpus | | Inflammation |
|  |  |  |  | Angular incisure | | HGIN |
| HGIN_3 | 71 | Female | Negative | Corpus | | Inflammation |
|  |  |  |  | Angular incisure | | HGIN |
| HGIN_4 | 55 | Male | Negative | Corpus | | Inflammation |
|  |  |  |  | Cardia | | HGIN |
| HGIN_5 | 58 | Male | Negative | Corpus | | Inflammation |
|  |  |  |  | Pyloric antrum | | HGIN |
| HGIN_6 | 77 | Female | Negative | Corpus | | Inflammation |
|  |  |  |  | Pyloric antrum | | HGIN |
| HGIN_7 | 62 | Female | Negative | Corpus | | Inflammation |
|  |  |  |  | Angular incisure | | HGIN |
| HGIN_8 | 65 | Female | Negative | Corpus | | Inflammation |
|  |  |  |  | Corpus | | HGIN |
| HGIN_9 | 54 | Male | Negative | Corpus | | Inflammation |
|  |  |  |  | Angular incisure | | HGIN |
| HGIN_10 | 69 | Male | Negative | Corpus | | Inflammation |
|  |  |  |  | Pyloric antrum | | HGIN |
| HGIN_11 | 54 | Male | Negative | Corpus | | Inflammation |
|  |  |  |  | Pyloric antrum | | HGIN |
| HGIN_12 | 66 | Male | Negative | Corpus | | Inflammation |
|  |  |  |  | Angular incisure | | HGIN |
| HGIN_13 | 73 | Female | NA | Corpus | | Inflammation |
|  |  |  |  | Corpus | | HGIN |
| HGIN_14 | 58 | Female | NA | Corpus | | Inflammation |
|  |  |  |  | Pyloric antrum | | HGIN |
| EGC_1 | 57 | Male | Negative | Corpus | | Inflammation |
|  |  |  |  | Corpus | | EGC |
| EGC_2 | 78 | Female | Negative | Corpus | | Inflammation |
|  |  |  |  | Pyloric antrum | | EGC |
| EGC_3 | 38 | Female | Negative | Pyloric antrum | | Inflammation |
|  |  |  |  | Corpus | | EGC |
| EGC_4 | 61 | Female | Positive | Corpus | | Inflammation |
|  |  |  |  | Angular incisure | | EGC |
| EGC_5 | 55 | Male | NA | Corpus | | Inflammation |
|  |  |  |  | Cardia | | EGC |
| EGC_6 | 59 | Female | NA | Corpus | | Inflammation |
|  |  |  |  | Cardia | | EGC |
| EGC_7 | 54 | Male | NA | Corpus | | Inflammation |
|  |  |  |  | Pyloric antrum | | EGC |
| EGC_8 | 57 | Male | NA | Corpus | | Inflammation |
|  |  |  |  | Corpus | | EGC |
| EGC_9 | 75 | Female | NA | Corpus | | Inflammation |
|  |  |  |  | Angular incisure | | EGC |
| EGC_10 | 40 | Female | NA | Corpus | | Inflammation |
|  |  |  |  | Angular incisure | | EGC |
| EGC_11 | 54 | Male | Negative | Corpus | | Inflammation |
|  |  |  |  | Corpus | | EGC |
| EGC_12 | 62 | Male | Negative | Corpus | | Inflammation |
|  |  |  |  | Corpus | | EGC |
| EGC_13 | 43 | Male | Positive | Corpus | | Inflammation |
|  |  |  |  | Pyloric antrum | | EGC |
| EGC_14 | 68 | Male | Negative | Corpus | | Inflammation |
|  |  |  |  | Corpus | | EGC |
| EGC_15 | 61 | Male | Negative | Corpus | | Inflammation |
|  |  |  |  | Corpus | | EGC |
| EGC_16 | 60 | Male | Negative | Pyloric antrum | | Inflammation |
|  |  |  |  | Pyloric antrum | | EGC |

**Table S2**. Sample information of the mRNA microarray

|  | **Sum**  **(*n* = 47)** | **Glgin-LGIN**  **(*n* = 17)** | **Ghgin-HGIN**  **(*n* = 14)** | **Gegc-EGC**  **(*n* = 16)** | ***P* value** | |
| --- | --- | --- | --- | --- | --- | --- |
| Gender |  |  |  |  |  | |
| Male | 23 (48.94%) | 6 (35.29%) | 8 (57.14%) | 9 (56.25%) | 0.3705* | |
| Female | 24 (51.06%) | 11 (64.71%) | 6 (42.86%) | 7 (43.75%) |
| Age (years) |  |  |  |  |  | |
| Median | 59 | 59 | 60 | 58 | 0.5524^†^ | |
| Range | 38–78 | 44–74 | 46–77 | 38–78 |  |  |

*Pearson’s chi-squared test.

^†^Kruskal–Wallis chi-squared test.

Glgin: paired gastritis control of LGIN; Ghgin: paired gastritis control of HGIN; Gegc: paired gastritis control of EGC.

| **Table S3. Mann–Whitney test of immune infiltration scores** | | | | | | |  |
| --- | --- | --- | --- | --- | --- | --- | --- |
|  | **EGC versus LGIN** | | **EGC versus HGIN** | | **HGIN versus LGIN** | | |
|  | ***P* value** | **FDR** | ***P* value** | **FDR** | ***P* value** | **FDR** | |
| B cells naive | 1.54E-05 | 5.31E-05 | 0.000611 | 0.001034 | 0.492781 | 0.832492 | |
| B cells memory | 2.73E-06 | 4.36E-05 | 0.000228 | 0.000456 | 0.597071 | 0.832492 | |
| Plasma cells | 0.000145 | 0.000213 | 0.030778 | 0.032243 | 0.444443 | 0.832492 | |
| T cells CD8 | 1.00E-04 | 0.000169 | 4.68E-05 | 0.000208 | 0.279439 | 0.683073 | |
| T cells CD4 naive | 5.56E-05 | 0.000102 | 2.17E-05 | 0.000207 | 0.149269 | 0.656783 | |
| T cells CD4 memory resting | 3.69E-05 | 7.38E-05 | 7.57E-05 | 0.000208 | 0.149269 | 0.656783 | |
| T cells CD4 memory activated | 0.000294 | 0.00036 | 0.000185 | 0.000406 | 0.652614 | 0.832492 | |
| T cells follicular helper | 1.93E-05 | 5.31E-05 | 5.97E-05 | 0.000208 | 0.335838 | 0.738844 | |
| T cells regulatory (Tregs) | 3.69E-05 | 7.38E-05 | 7.57E-05 | 0.000208 | 0.652614 | 0.832492 | |
| T cells gamma delta | 2.41E-05 | 5.88E-05 | 2.82E-05 | 0.000207 | 0.543719 | 0.832492 | |
| NK cells resting | 0.000248 | 0.000321 | 7.57E-05 | 0.000208 | 0.200105 | 0.683073 | |
| NK cells activated | 0.000412 | 0.000453 | 2.17E-05 | 0.000207 | 0.099995 | 0.656783 | |
| Monocytes | 0.000208 | 0.000286 | 0.00243 | 0.003145 | 0.262117 | 0.683073 | |
| Macrophages M0 | 0.003685 | 0.003685 | 0.009064 | 0.010495 | 0.891131 | 0.891131 | |
| Macrophages M1 | 1.93E-05 | 5.31E-05 | 0.000185 | 0.000406 | 0.829677 | 0.869186 | |
| Macrophages M2 | 0.000145 | 0.000213 | 0.00243 | 0.003145 | 0.799303 | 0.869186 | |
| Dendritic cells resting | 1.54E-05 | 5.31E-05 | 0.002069 | 0.003035 | 0.245529 | 0.683073 | |
| Dendritic cells activated | 1.54E-05 | 5.31E-05 | 0.000417 | 0.000765 | 0.68113 | 0.832492 | |
| Mast cells resting | 4.62E-06 | 4.36E-05 | 0.047205 | 0.047205 | 0.048385 | 0.656783 | |
| Mast cells activated | 5.94E-06 | 4.36E-05 | 0.027513 | 0.030264 | 0.099995 | 0.656783 | |
| Eosinophils | 0.000412 | 0.000453 | 0.001053 | 0.001654 | 0.829677 | 0.869186 | |
| Neutrophils | 0.002162 | 0.002265 | 0.009064 | 0.010495 | 0.492781 | 0.832492 | |

| **Table S4. Twenty-two coDEGs from merging four groups of DEGs** | |
| --- | --- |
| **Co-up DEGs** | **Co-down DEGs** |
| *TIMP1* | *GADD45B* |
| *HENMT1* | *GPER1* |
| *CXCL16* | *DUOX1* |
| *SKP2* | *GSTM2* |
| *LAMP3* | *PGA4* |
| *RARRES1* | *MST1* |
| *MANEAL* | *GSTA4* |
| *PLEKHS1* | *SSC5D* |
| *PKDCC* | *LAMB2* |
| *DYX1C1* | *ADAMTS13* |
| *KCNE3* | *RGN* |

| **Table S5. Correlations between DEGs and stem scores** | | | | | | |
| --- | --- | --- | --- | --- | --- | --- |
| **Up genes** | **cor** | ***P* value** |  | **Down genes** | **cor** | ***P* value** |
| *GRIN2D* | 0.556 | 6.18E-09 |  | *BCL2L11* | −0.561 | 3.99E-09 |
| *BRCA1* | 0.755 | 1.61E-18 |  | *RET* | −0.442 | 8.40E-06 |
| *TIMP1* | 0.413 | 3.46E-05 |  | *ALB* | −0.51 | 1.33E -07 |
| *HENMT1* | 0.62 | 2.74E-11 |  | *GADD45B* | −0.647 | 1.82E-12 |
| *CXCL16* | 0.442 | 8.07E-06 |  | *GPER1* | −0.55 | 9.56E-09 |
| *SKP2* | 0.768 | 1.55E-19 |  | *DUOX1* | −0.454 | 4.33E-06 |
| *LAMP3* | 0.477 | 1.16E-06 |  | *GSTM2* | −0.542 | 1.74E-08 |
| *RARRES1* | 0.531 | 3.60E-08 |  | *PGA4* | −0.545 | 1.35E-08 |
| *MANEAL* | 0.765 | 2.84E-19 |  | *MST1* | −0.539 | 2.07E-08 |
| *PLEKHS1* | 0.554 | 7.16E-09 |  | *GSTA4* | −0.499 | 3.07E-07 |
| *PKDCC* | 0.539 | 2.15E-08 |  | *SSC5D* | −0.575 | 1.38E-09 |
| *DYX1C1* | 0.535 | 2.82E-08 |  | *LAMB2* | −0.379 | 0.000163 |
| *KCNE3* | 0.51 | 1.52E-07 |  | *ADAMTS13* | −0.748 | 4.63E-18 |
|  |  |  |  | *RGN* | −0.499 | 3.01E-07 |

**Table S6. Univariate and multivariate Cox proportional hazard regression analysis of overall survival and disease-free survival in training (*n* = 547), testing (*n* = 406), GSE62254 (*n* = 300), and GSE15460 (*n* = 248) patients according to the five-gene signature, gender, age, and stage**

| **Variables** | **Univariate analysis** | | | | **Multivariate analysis** | | | | |
| --- | --- | --- | --- | --- | --- | --- | --- | --- | --- |
|  | **HR (95% CI)** | | ***P* value** | | | **HR (95% CI)** | | | ***P* value** |
| Training dataset |  | |  | | | |  | |  |
| Overall survival |  | |  | | | |  | |  |
| Risk score (high/low) | 2.36 (1.86–3.00) | | 2.26 × 10^−12^*** | | | | 1.94 (1.52–2.48) | | 9.26 × 10^−8^*** |
| Gender M/F | 0.98 (0.76–1.26) | | 0.871 | | | | 0.94 (0.73–1.21) | | 0.651 |
| Age | 1.01 (0.995–1.02) | | 0.293 | | | | 1.02 (1.01–1.03) | | 0.0031** |
| Stage II/I | 2.49 (1.25–4.94) | | 0.0092** | | | | 2.46 (1.24–4.90) | | 0.010* |
| Stage III/I | 6.18 (3.22–11.84) | | 4.07 × 10^−8^*** | | | | 5.99 (3.11–11.51) | | 8.28 × 10^−8^*** |
| Stage IV/I | 13.63 (7.10–26.15) | | 4.03 × 10^−15^*** | | | | 13.21 (6.84–25.49) | | 1.42 × 10^−14^*** |
| Disease-free survival |  | |  | | | |  | |  |
| Risk score (high/low) | 2.64 (1.85–3.77) | | 8.40 × 10^−8^*** | | | | 2.27 (1.57–3.29) | | 1.24 × 10^−5^*** |
| Gender M/F | 0.97 (0.67–1.40) | | 0.860 | | | | 1.34 (0.91–1.97) | | 0.132 |
| Age | 1.00 (0.99–1.02) | | 0.676 | | | | 1.01 (1.00–1.03) | | 0.103 |
| Stage II/I | 2.28 (0.68–7.62) | | 0.181 | | | | 1.87 (0.56–6.29) | | 0.310 |
| Stage III/I | 5.48 (1.70–17.65) | | 0.0044** | | | | 4.49.(1.38–14.56) | | 0.012* |
| Stage IV/I | 13.55 (4.22–43.47) | | 1.17 × 10^−5^*** | | | | 11.40 (3.52–36.94) | | 4.93 × 10^−5^*** |
| Independent testing dataset (TCGA STAD) | |  | |  | | | |  | |
| Overall survival |  | |  | | | |  | |  |
| Risk score (high/low) | 1.43 (1.04–1.98) | | 0.0289* | | | | 1.57 (1.13–2.17) | | 0.00706** |
| Gender M/F | 1.19 (0.84–1.68) | | 0.324 | | | | 1.12 (0.79–1.58) | | 0.530 |
| Age | 1.02 (1.00–1.04) | | 0.0099** | | | | 1.03 (1.01–1.05) | | 2.28 × 10^−4^*** |
| Stage II/I | 1.44 (0.75–2.78) | | 0.276 | | | | 1.47 (0.76–2.84) | | 0.252 |
| Stage III/I | 2.26 (1.23–4.16) | | 0.0088** | | | | 2.52 (1.36–4.65) | | 0.0032** |
| Stage IV/I | 4.03 (2.01–8.06) | | 8.38 × 10^−5^*** | | | | 5.57 (2.73–11.36) | | 2.39 × 10^−6^*** |
| Disease-free survival |  | |  | | | |  | |  |
| Risk score (high/low) | 1.83 (1.25–2.69) | | 0.00203** | | | | 1.92 (1.30–2.83) | | 0.00103** |
| Gender M/F | 2.03 (1.28–3.22) | | 0.0027** | | | | 1.97 (1.23–3.13) | | 0.00446** |
| Age | 1.00 (0.98–1.02) | | 0.818 | | | | 1.01 (0.99–1.02) | | 0.574 |
| Stage II/I | 1.72 (0.85–3.51) | | 0.133 | | | | 1.67 (0.82–3.41) | | 0.156 |
| Stage III/I | 2.03 (1.03–4.02) | | 0.00415** | | | | 1.97 (0.99–3.92) | | 0.0525 |
| Stage IV/I | 3.50 (1.55–7.90) | | 0.0026** | | | | 3.64 (1.59–8.32) | | 0.00223** |
| GSE15460 |  | |  | | | |  | |  |
| Overall survival |  | |  | | | |  | |  |
| Risk score (high/low) | 1.80 (1.25–2.57) | | 0.0014** | | | | 1.48 (1.03–2.12) | | 0.0355* |
| Gender M/F | 1.11 (0.76–1.61) | | 0.595 | | | | 0.71 (0.48–1.06) | | 0.0907 |
| Age | 1.00 (0.98–1.01) | | 0.719 | | | | 1.00 (0.99–1.02) | | 0.578 |
| Stage II/I | 3.27 (1.18–9.07) | | 0.0232* | | | | 3.16 (1.14–8.77) | | 0.0276* |
| Stage III/I | 9.80 (3.90–24.63) | | 1.23 × 10^−6^*** | | | | 9.91 (3.93–25.02) | | 1.20 × 10^−6^*** |
| Stage IV/I | 19.79 (7.77–50.37) | | 3.84× 10^−10^*** | | | | 20.95 (8.14–53.93) | | 2.89 × 10^−10^*** |
| GSE62254 |  | |  | | | |  | |  |
| Overall survival |  | |  | | | |  | |  |
| Risk score (high/low) | 2.85 (2.06–3.93) | | 2.02 × 10^-10^*** | | | | 2.49 (1.78–3.48) | | 8.05 × 10^-8^* |
| Gender M/F | 0.90 (0.65–1.27) | | 0.559 | | | | 1.16 (0.82–1.64) | | 0.406 |
| Age | 1.01 (1.00–1.03) | | 0.181 | | | | 1.03 (1.01–1.04) | | 0.578 |
| Stage II/I | 1.93 (0.75–4.97) | | 0.172 | | | | 1.57 (0.61–4.08) | | 0.351 |
| Stage III/I | 4.01 (1.60–10.03) | | 0.00305** | | | | 3.36 (1.33–8.50) | | 0.0104* |
| Stage IV/I | 9.73 (3.90–24.25) | | 1.04 × 10^−6^*** | | | | 8.26 (3.27–20.84) | | 7.72 × 10^−6^*** |

**p* < 0.05; ***p* < 0.01; ****p* < 0.001.

HR: hazard ratio; CI: confidence interval.
